# Genome-wide association study reveals novel genomic regions governing agronomic and grain quality traits and superior allelic combinations for Basmati rice improvement

**DOI:** 10.3389/fpls.2022.994447

**Published:** 2022-12-05

**Authors:** Krishnan P. Abhijith, S. Gopala Krishnan, Kuram Tirumala Ravikiran, Gaurav Dhawan, Pankaj Kumar, Kunnummal Kurungara Vinod, Prolay Kumar Bhowmick, Mariappan Nagarajan, Rakesh Seth, Ritesh Sharma, Sourav Kumar Badhran, Haritha Bollinedi, Ranjith Kumar Ellur, Ashok Kumar Singh

**Affiliations:** ^1^ Division of Genetics, ICAR-Indian Agricultural Research Institute, New Delhi, India; ^2^ Rice Breeding and Genetics Research Centre, ICAR-Indian Agricultural Research Institute, Aduthurai, Tamil Nadu, India; ^3^ Regional Station, ICAR-Indian Agricultural Research Institute, Karnal, Haryana, India; ^4^ Basmati Export Development Foundation (BEDF), Meerut, Uttar Pradesh, India; ^5^ IARI-Collaborative Outstation Research Centre (CORC), Patiala, Punjab, India

**Keywords:** Basmati, GWAS, Hybrid, MTA, rice, SNP, BLINK

## Abstract

**Background:**

Basmati is a speciality segment in the rice genepool characterised by explicit grain quality. For the want of suitable populations, genome-wide association study (GWAS) in Basmati rice has not been attempted.

**Materials:**

To address this gap, we have performed a GWAS on a panel of 172 elite Basmati multiparent population comprising of potential restorers and maintainers. Phenotypic data was generated for various agronomic and grain quality traits across seven different environments during two consecutive crop seasons. Based on the observed phenotypic variation, three agronomic traits namely, days to fifty per cent flowering, plant height and panicle length, and three grain quality traits namely, kernel length before cooking, length breadth ratio and kernel length after cooking were subjected to GWAS. Genotyped with 80K SNP array, the population was subjected to principal component analysis to stratify the underlying substructure and subjected to the association analysis using Bayesian-information and Linkage-disequilibrium Iteratively Nested Keyway (BLINK) model.

**Results:**

We identified 32 unique MTAs including 11 robust MTAs for the agronomic traits and 25 unique MTAs including two robust MTAs for the grain quality traits. Six out of 13 robust MTAs were novel. By genome annotation, six candidate genes associated with the robust MTAs were identified. Further analysis of the allelic combinations of the robust MTAs enabled the identification of superior allelic combinations in the population. This information was utilized in selecting 77 elite Basmati rice genotypes from the panel.

**Conclusion:**

This is the first ever GWAS study in Basmati rice which could generate valuable information usable for further breeding through marker assisted selection, including enhancing of heterosis.

## 1 Introduction

Rice (*Oryza sativa* L.) is cultivated across the world and is a staple food for the majority of the population in Asia and Africa. Basmati rice is a prominent subgroup of rice, indigenous to the Indian subcontinent, which is endowed with a unique combination of aroma, grain characteristics and palatability, due to which it is a premium price in both domestic and international markets (Singh et al., 1988). During 2020-21, the earnings through the export of Basmati rice was 298 billion rupees to foreign markets (APEDA, 2022). Basmati belongs to a narrow group within the rice gene pool (Glaszmann, 1987), where the productivity is comparatively lower than the other major rice groups. Being commercially important and grown in a limited area under geographical indication, improvement of Basmati rice production is possible only through improving productivity. Therefore, it is important to develop superior lines without jeopardizing the standards for Basmati grain and cooking quality while maintaining sufficient genetic variability within the breeding pool for further improvement of Basmati rice. Besides the development of pureline varieties, the development of the Basmati hybrids is an alternative for enhancing its productivity. The development of a population that comprises elite but diverse breeding lines can also serve as a source population for developing hybrid breeding-oriented elite germplasm. Unprecedented developments in sequencing technologies have enabled the generation and utilization of valuable genomic information from these populations, in order to address various challenges associated with crop improvement (Varshney et al., 2014).

Genome-wide association study (GWAS) is a very popular quantitative genomics tool, which has gained prominence in mapping quantitative traits with the advent of high-density genotyping platforms, like SNP arrays. GWAS has played a pivotal role in identifying many important genes which govern various complex agronomic traits such as flowering time, plant height and panicle length in rice (Begum et al., 2015; Yano et al., 2016; Reig-Valiente et al., 2018; Zhang et al., 2019; Zhou and Huang, 2019; Verma et al., 2021), particularly using collections of unrelated diverse germplasm (Huang et al., 2012; Qiu et al., 2021; Ravikiran et al., 2022). Although GWAS is helpful in mining novel alleles for complex traits, it requires further validation in breeding populations for its usefulness in marker-assisted breeding (Begum et al., 2015). Therefore, performing association studies in adapted breeding lines has been suggested as a better alternative, where the results can be directly applied in ongoing breeding programs (Zhang et al., 2014). Elite breeding lines at the end of the selection cycle have proven to be potential candidates for GWAS (Bordes et al., 2014; Begum et al., 2015). It can also assist in effecting strategically planned crosses so as to ensure that there is the retention of sufficient genetic diversity for target traits in the superior performing populations after each selection cycle.

Among the agronomic traits in rice, grain quality is of paramount importance in defining consumer acceptance and marketability. In premium quality rice such as Basmati, where quality enjoys equal, sometimes even more weightage than grain yield, traits such as kernel length before cooking, kernel length after cooking, and length-breadth ratio are considered major physical quality traits that largely determine consumer preference. Several studies have reported the allelic variations for kernel length before cooking, and the length-breadth ratio in rice through GWAS (Ponce et al., 2020; Qiu et al., 2021). Employing GWAS, Misra et al. (2017) identified MTAs associated with cooked and raw grain length, width and shape. However, there has been limited efforts on association mapping for kernel length after cooking.

Several statistical models such as general linear model (GLM) and mixed linear model (MLM), have been developed for computational analysis in GWAS. Multiple loci models such as multi-locus mixed model (MLMM), Fixed and random model circulating probability unification (FarmCPU), and Bayesian-information and linkage-disequilibrium iteratively nested keyway (BLINK) are considered superior over single-locus models such as GLM and MLM (Tibbs Cortes et al., 2021), as they offer better computing efficiency, more statistical power and lesser computational burden (Huang et al., 2019). Among the latest models for GWAS, BLINK is preferred over other models, since it has higher computational efficiency and power to detect marker-trait associations (Wang and Zhang, 2021). BLINK uses Bayesian information content (BIC) in a fixed-effect model and replaces the bin approach used in the FarmCPU model with linkage disequilibrium (LD, Huang et al., 2019).

Limited efforts have been made towards improvement of Basmati rice parental lines. A set of 172 elite Basmati rice breeding lines have been developed through systematic intermating within restorers and maintainers. The utility of these elite breeding lines requires the characterisation of accumulated genomic regions associated with target traits. To address this, GWAS was performed on a collection of 172 elite Basmati breeding lines, which were bred for developing diverse parental lines for Basmati hybrid breeding. Mapping was carried out on six agronomic and grain quality-related traits by evaluating them across seven different environments, to explore the allelic status of these traits and to identify breeding lines with superior allelic combinations in the population.

## 2 Materials and methods

### 2.1 Plant material and field experiment

An association mapping panel comprising of 172 diverse Basmati breeding lines developed and maintained at the Division of Genetics, ICAR-Indian Agricultural Research Institute (ICAR-IARI), New Delhi, India was constituted for this study. These breeding lines were derived from multi-parent crosses involving 15 Basmati rice and 9 non-Basmati rice genotypes as founder parents (**Supplementary Table 1**). Based on the presence of fertility restorer genes, *Rf3* and *Rf4*, the panel accommodated 119 putative restorers and 53 putative maintainers. The complete set of genotypes was evaluated at three Basmati growing locations in India during the *Kharif* season of 2019, *viz.* ICAR-IARI Research Station, Karnal, Haryana located at 29.50°N, 77.10°E, 243m (KNL19), IARI-CORC, Rakhra, Punjab, located at 30.36°N, 76.98°E, 252m (RKR19), and Basmati Export and Development Foundation, Modipuram, Uttar Pradesh located at 29.04°N, 77.42°E, 230m (MDP19) and at four locations during *Kharif* 2020 i.e., ICAR-IARI New Delhi located at 28.08°N, 77.12°E, 228.6m (DEL20) in addition to the previous season’s three locations (KNL20, RKR20 and MDP20). The seeds were sown in nursery beds and 25-30 days old seedlings were transplanted manually in puddled experimental plots. The experimental design followed was augmented RCB with 6 blocks, in all the locations. Genotypes were planted in 1 m^2^ plots each with 20 cm spacing between rows and 15 cm between plants. Except for time of sowing, all the environments received similar and recommended agronomic practices for irrigated transplanted rice.

### 2.2 Phenotyping for agro-morphological traits

The pre-harvest observations included days to 50% flowering (DFF), plant height (PH), and panicle length (PL). DFF was recorded on single plot basis, while for the remaining traits five randomly selected plants per genotype were tagged after flowering and data were recorded at physiological maturity. Data from all the seven environments were used for the statistical analyses. Being Basmati lines, the genotypes were also assessed for grain and cooking quality parameters. For this, harvested grains were left for ageing for up to 3 months, and about 200g per genotype was dehusked using SATAKE™ testing husker (Model THU35B, Satake Corporation, Hiroshima, Japan) and later milled through SATAKE™ testing mill (Model TM05, Satake Corporation, Hiroshima, Japan). The observations were recorded on kernel length before cooking (KLBC) and length-breadth ratio (LBR) was calculated by dividing KLBC by kernel breadth. The milled samples of each entry were then cooked for estimating kernel length after cooking (KLAC). A total of ten whole milled grains were soaked in a test tube containing 10ml of distilled water for 30 minutes and then placed in a water bath containing boiling water for 8 to 10 minutes (Khanna et al., 2015).

### 2.3 Statistical analysis

We have used the data from the four environments (DEL20, KNL20, RKR20, and MDP20) for the GWAS. The data from individual environments were analyzed separately by adopting a linear mixed model approach. In each environment, block effects were considered random, while the genotype effects were considered fixed. Best linear unbiased estimates (BLUEs) were calculated for the augmented complete block design (ACBD) using PBTools v.1.3 (IRRI, 2014) software for individual environments. The model used here is


$${\text{Y}}_{\text{ij}}=\text{ µ }+{\text{ Gen}}_{\text{j}}+{\text{ Check}}_{\text{j}}+{\text{ Block}}_{\text{i}}+{\text{ e}}_{\text{ij}}$$

Where Y_ij_ denotes the phenotypic value of the i^th^ genotype at the j^th^ environment, µ is the mean effect, Gen_j_ stands for the fixed effect of unreplicated genotypes, Block_i_ is representing the fixed effect of the i^th^ block, and Check_j_ denotes the effect of the checks that within each block, and the factor e_ij_ is representing the error component.

Best linear unbiased estimates (BLUE) of genotypes were extracted from the mixed model which was used for further downstream analyses.

### 2.4 DNA isolation and SNP genotyping

Genomic DNA was isolated from the young leaves at the seedling stage using CTAB method (Murray and Thompson, 1980). The quality of the isolated DNA samples was initially checked on 0.8% agarose gel followed by further quantification using a nanodrop spectrophotometer (NanoDropTM 2000/2000c, Thermo Fisher Scientific, DE, United States). The samples were genotyped using an 80K Rice Pan-Genome Genotyping Array (RPGA) (Daware et al., 2022). Briefly, the RPGA harbours evenly dispersed 80504 SNPs, including 20478 SNPs from 12 pseudo-chromosomes of 3K rice pan-genome.

### 2.5 Population structure and linkage disequilibrium decay

For the final analysis, the SNPs from the pseudochromosomes were removed, leaving 60026 SNPs corresponding to the 12 Nipponbare chromosomes. The data were further filtered using TASSEL 5.2.81 software (Bradbury et al., 2007) through a couple of filters i.e., markers with a minor allele frequency cutoff of 5%, and with over 10% of missing reads, leaving the final number to 31701 markers. For analysing the population structure, markers that are close together and are in LD were removed, for which *SNPRelate* package was used under R statistical environment with a 100Kb window size and r^2^ threshold of 0.4 (Zheng, 2018). Resulting 5003 pruned SNP markers were then used for the analysis with STRUCTURE software v2.3.4 (Pritchard et al., 2010). The optimum number of subpopulations was assessed by testing for K=1 to 6 using five independent runs of 100,000 burn-ins followed by 100,000 iterations using a model allowing for admixture and correlated allele frequencies. The optimum number of K was determined according to the method given by Evanno et al. (2005) using an online tool, ‘Structure Harvester’ (Earl and vonHoldt, 2012) by plotting the *ad hoc* statistic (ΔK) against the natural logarithms of probability data [LnP(K)].

Pairwise LD between 31701 SNP markers were assessed by estimating r^2^ values in TASSEL 5.2.81 software. The r^2^ values of intrachromosomal marker pairs were then filtered out using criteria, p< 0.05 for the LD decay analysis. Marker pairs within 5Kb distances are then clustered together in individual bins. LD decay curve was created by plotting the average r^2^ values of bins against the distance. The corresponding distance at which the average r^2^ value dropped to half of its average maximum value was considered the rate of LD decay (Huang et al., 2012).

### 2.6 Association mapping

The marker data comprising 31701 SNPs were analysed by implementing a multilocus model, BLINK which was executed in GAPIT v.3 (Wang and Zhang, 2021). The BLUEs for various traits generated across environments were separately used for the analysis. The standard Bonferroni threshold (Rice et al., 2008) was used to determine the significant marker-trait associations (MTA) for avoiding false positives. Specific quantitative trait nucleotide (QTN) was identified for every significant MTAs from the GWAS result. QTNs which were consistently found in more than one environment were considered for identifying stable MTAs. These MTAs were further investigated for average phenotypic values of their alternate alleles. Furthermore, for the traits where more than one such stable MTAs were observed, we have estimated the phenotypic effect of the different allelic combinations in the population. The alleles which enhanced the phenotypic effect were considered positive alleles. The average effect of both alleles of each MTA was compared, and their significance was tested using a single factor ANOVA. Finally, an upset plot was created using the package *upsetR* to select best allelic combinations and the individuals possessing them (Conway et al., 2017).

### 2.7 Assessment of the novelty of identified MTAs

The MTAs which were detected in more than one environment were considered for assessing their novelty. Physical positions of such stable MTAs were compared with previously reported quantitative trait loci (QTL) and candidate genes for the concerned trait. The QTLs in the vicinity of stable MTAs were searched using the Gramene QTL database (https://www.gramene.org/) and QTL annotation rice online database (QTARO, http://qtaro.abr.affrc.go.jp/), coupled with a thorough literature search. Putative candidate genes in the vicinity of MTAs were browsed in the database of Rice Annotation Project Database (RAP-DB, https://rapdb.dna.affrc.go.jp/).

## 3 Results

### 3.1 Phenotypic variation for agronomic and grain quality attributes in the association panel

**Figure 1:**
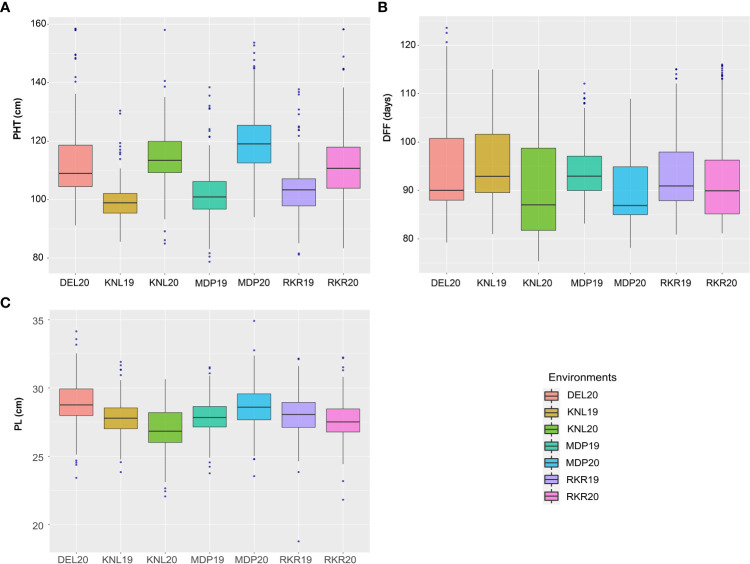
Boxplots depicting the distribution of **(A)** Plant height (cm), **(B)** Days to fifty per cent flowering and **(C)** Panicle length (cm) across seven environments.

**Figure 2:**
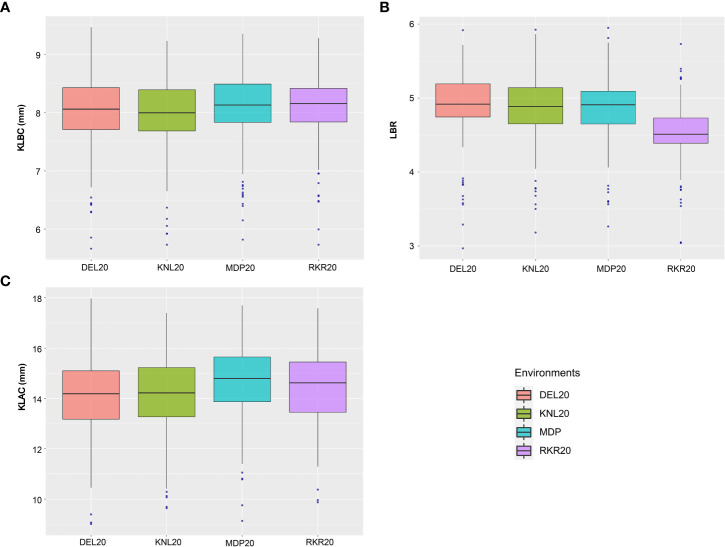
Boxplots depicting the distribution of **(A)** KLBC (mm), **(B)** LBR and **(C)** KLAC (mm) across across environments.

The analysis of variance (ANOVA) of individual environments revealed significant variability for all the traits among the genotypes, except for PL (**Supplementary Table 2**). These results indicate the existence of a substantial amount of variation for these traits in the panel. Further, Spearman’s rank correlation between the environments showed high significance for all the traits studied (**Supplementary Table 3**). PHT displayed a wider variation in its expression across environments (**Table 1**). The lowest plant height was recorded by the entry, GPM115 (78.74 cm) in MDP19 and the highest plant height was observed for GPR47 (158.43 cm) at DEL20 (**Supplementary Table 4**). The box plot of PHT indicated significant variation for plant height across all environments except in KNL19. The PH of the genotypes in *Kharif* 2019 were generally shorter coupled with a lesser range than that of *Kharif* 2020 (**Figure 1**). A similar trend was also seen for the distribution of DFF. The genotypes flowered late in *Kharif* 2019 compared to that of *Kharif* 2020. In case of DFF, in all the locations except in MDP20, the entries registered considerable variation for flowering. The largest variation was seen in KNL20 with a CV of 12.2%. Further, the distribution of genotypes, in general, was skewed towards late maturity across the environments. In the case of PL, however, the seasonal variation across locations was less pronounced. The lowest value of panicle length was registered in RKR19 for GPM77 (18.77 cm) whilst the highest value was recorded for GPM100 (34.91 cm) in MDP20. The pattern of variation for the grain quality traits, was more or less uniform across all the environments for all three traits (**Figure 2**). The highest variation for KLBC was seen at MDP20 with a CV of 8.35% and for KLAC and LBR, highest CV was at DEL20 (11.18%) and KNL20 (9.52%), respectively (**Table 2**). KLBC ranged between 5.66 mm at DEL20 for GPR43 and 9.47 mm for GPR308 at DEL20. In the case of LBR, the lowest and highest values were recorded at DEL20 (2.97) and MDP20 (5.95), respectively with the lowest mean (4.53) observed at RKR20 (**Supplementary Table 5F**). For KLAC, lowest value of 9.01 mm was observed for GPR7 at DEL20 while GPR259 showed the highest value (17.59 mm) at DEL20.

**Table 1:** Summary statistics of three agronomic traits recorded on 172 breeding lines evaluated across seven environments.

Trait	Location	Min	Max	Mean (±SE)	Standard deviation	CV (%)
**PHT (cm)**	DEL20	91.19	158.43	112.87 ± 1.07	14.01	12.41
	KNL19	85.59	130.42	99.26 ± 0.56	7.25	7.3
	KNL20	84.99	158.04	114.42 ± 0.77	10.16	8.88
	MDP19	78.74	138.43	101.97 ± 0.75	9.92	9.73
	MDP20	94.05	153.7	119.64 ± 0.85	11.08	9.26
	RKR19	81.21	137.73	103.32 ± 0.75	9.79	9.48
	RKR20	83.37	158.25	112.05 ± 0.96	12.61	11.25
**DFF (days)**	DEL20	79.25	123.59	94.09 ± 0.74	9.69	10.3
	KNL19	80.97	115.01	95.8 ± 0.65	8.51	8.88
	KNL20	75.37	114.96	90.98 ± 0.85	11.1	12.2
	MDP19	83.16	112.07	94.51 ± 0.48	6.34	6.71
	MDP20	78.12	108.93	90.05 ± 0.56	7.36	8.17
	RKR19	80.87	115.03	93.49 ± 0.69	9.03	9.66
	RKR20	81.12	116	92.77 ± 0.76	10.02	10.8
**PL (cm)**	DEL20	23.42	34.15	28.85 ± 0.13	1.73	5.98
	KNL19	23.84	31.91	27.84 ± 0.1	1.37	4.91
	KNL20	22.06	30.64	26.97 ± 0.13	1.69	6.25
	MDP19	23.75	31.52	27.87 ± 0.1	1.31	4.72
	MDP20	23.54	34.91	28.57 ± 0.14	1.77	6.19
	RKR19	18.77	32.15	28 ± 0.12	1.56	5.56
	RKR20	21.82	32.23	27.62 ± 0.12	1.59	5.76

Min, Minimum; Max, Maximum; SE, Standard Error; CV, Coefficient of variation; DFF, Days to fifty percent flowering; PHT, Plant height; PL, Panicle length.

**Table 2:** Summary statistics of three, grain quality traits recorded on 172 breeding lines evaluated across four environments.

Grain quality trait	Location	Min	Max	Mean (± SE)	Standard deviation	CV (%)
**KLBC (mm)**	DEL20	5.66	9.47	7.99 ± 0.05	0.65	8.16
	KNL20	5.73	9.24	7.96 ± 0.05	0.65	8.2
	MDP20	5.82	9.36	8.1 ± 0.05	0.68	8.35
	RKR20	5.73	9.28	8.06 ± 0.04	0.56	7.01
**KLAC (mm)**	DEL20	9.01	17.97	14.08 ± 0.12	1.57	11.18
	KNL20	9.64	17.4	14.12 ± 0.12	1.58	11.15
	MDP20	9.13	17.7	14.62 ± 0.11	1.49	10.21
	RKR20	9.87	17.58	14.49 ± 0.11	1.49	10.29
**LBR**	DEL20	2.97	5.92	4.9 ± 0.03	0.45	9.26
	KNL20	3.18	5.92	4.88 ± 0.04	0.46	9.52
	MDP20	3.26	5.95	4.87 ± 0.03	0.44	9.13
	RKR20	3.04	5.73	4.53 ± 0.03	0.37	8.13

Min, Minimum; Max, Maximum; SE, Standard Error; CV, Coefficient of variation; KLBC, Kernel length before cooking; LBR, Length-Breadth Ratio; KLAC, Kernel length after cooking.

### 3.2 The association panel is composed of two sub-populations

**Figure 3:**
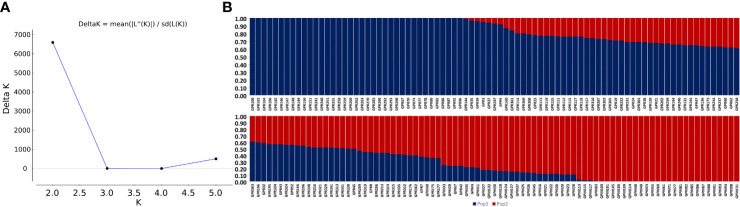
**(A)** ΔK plot depicting two subgroups in the population by Evanno’s method. The highest ΔK was at K = 2, **(B)** the bar plot showing membership fractions of the genotypes in the two sub-populations identified.

**Figure 4:**
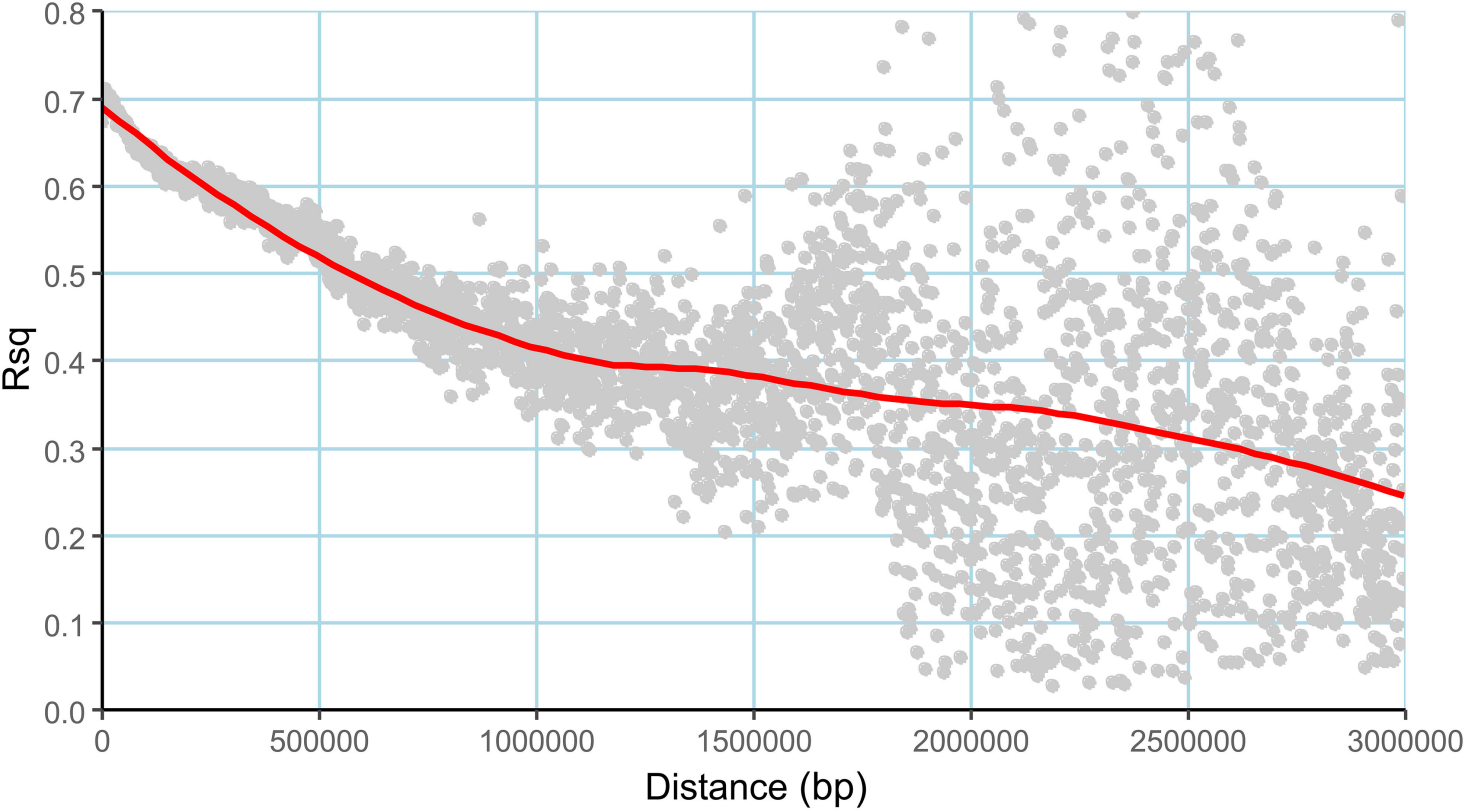
Linkage disequilibrium (LD) decay plot of the association mapping panel, the average r^2^ values (Rsq) plotted against the distance (Dist) in base pairs (bp).

The admixture model-based simulation using genome wide markers revealed a sharp Δk peak when k was two (**Figure 3A**), implying the existence of two subpopulations. The subpopulations are denoted as subpopulation1 (SP1) and subpopulation 2 (SP2). The genotypes exhibiting ≥80% likelihood are designated to specific subpopulations whereas others are categorized as admixtures (Famoso et al., 2011). Both the subpopulations were accommodating an almost equal number of breeding lines and are stratified with Fst values of 0.72 and 0.20 respectively. The SP1 included 48 individuals and SP2 comprised 47 individuals. The remaining 77 individuals were considered admixtures (**Figure 3B**). The pair-wise LD between markers as average r^2^ values of bins were plotted against the physical distance between the markers, resulted in a near flat curve indicating relatively less LD decay in the population. The maximum r^2^ value (r^max^) obtained was ~0.7 within a genomic span of less than 5Kb region and the r^max^ was reduced to half at a distance of ~1.8 Mb (**Figure 4**).

### 3.3 Marker-trait associations detected for agronomic and grain quality traits

**Figure 5:**
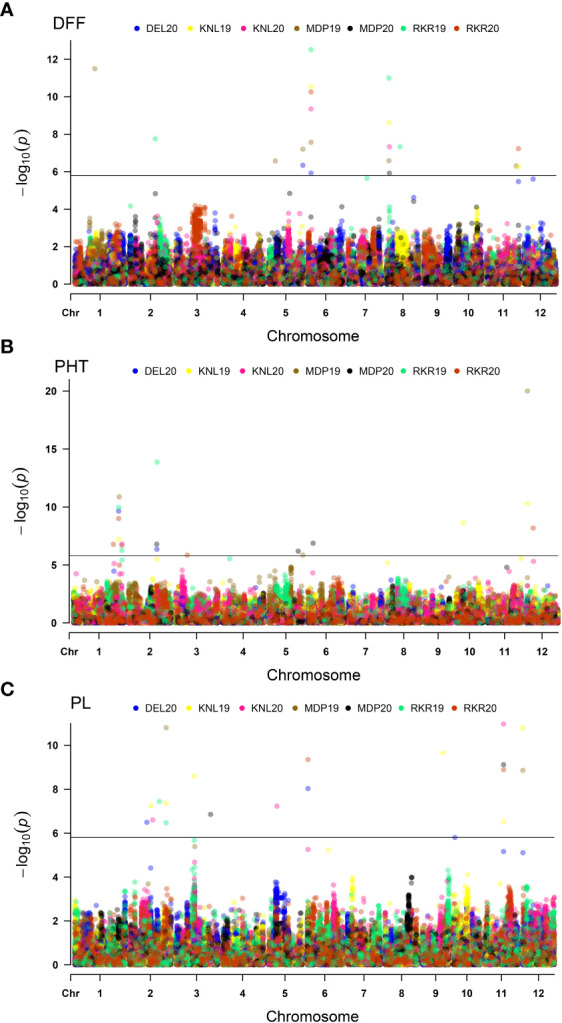
Multi-Environmental Manhattan plots depicting significant MTAs for various agronomic traits *viz*., Days to fifty percent flowering (DFF) **(A)**, Plant height (PHT) **(B)** and Panicle length (PL) **(C)**. The dots marked in different colours indicate QTNs detected in different environments. The horizontal line indicates Bonferoni cut-off.

Association analysis using the BLINK model, used a threshold *p* cutoff of <1.577E-06 based on Bonferroni estimate (**Figure 5**). A total of 56 MTAs including 32 unique MTAs were detected for three agronomic traits (DFF, PHT and PL) across seven environments as summarized in **Table 3**. In the case of DFF, 19 MTAs were detected, including nine unique ones, of which one quantitative trait nucleotide (QTN) each was present on chromosomes 1, 2, 6 and 11. Two MTAs were present on chromosome 5 and three MTAs were detected on chromosome 8. Considering the MTAs detected in more than one environment as stable, the analysis has detected five stable MTAs for DFF. These are named as *qDFF5.1*, *qDFF6.1*, *qDFF8.1*, *qDFF8.2* and *qDFF11.1*. The most stable MTA was *qDFF6.1* detected in six out of seven environments. Among the 19 QTNs identified for PHT, 13 were unique and were distributed across six different chromosomes. Chromosome 1 carried four unique MTAs, chromosome 2 had three, chromosomes 5 and 12 had two MTAs each and chromosomes 6 and 10 possessed one unique MTA each. The SNP, AX154964191 (*qPHT1.1*) was detected in four environments and hence regarded as the most stable MTA for PHT. Other stable MTAs for PHT were *qPHT1.2*, *qPHT12.1*. For PL, 18 MTAs were detected spanning across eight different chromosomes and six environments, of which 13 were unique. Chromosomes 5, 10, 11 and 12 carried one MTA each, while chromosomes 3 and 6 each carried two unique MTAs. However, there were five MTAs located on chromosome 2. The SNP, AX182222834 (*qPL11.1*) was detected across four environments and hence was considered the most stable MTA for PL. Besides, two more MTAs, *qPL2.4* and *qPL6.1* were also identified as stable.

**Table 3:** Marker trait associations (MTA) detected in GWAS by using BLINK model for the agronomic traits in seven environments.

Trait	Environment	SNP	Chromosome	Position	P value	MTA
DFF	DEL20	AX182223369	5	29587984	4.54E-07	*qDFF5.1*
	DEL20	AX154828491	6	2927622	1.18E-06	*qDFF6.1*
	KNL19	AX154828491	6	2927622	2.89E-11	*qDFF6.1*
	KNL19	AX123164507	8	2208785	2.46E-09	*qDFF8.1*
	KNL19	AX117352818	11	27605483	5.51E-07	*qDFF11.1*
	KNL20	AX154828491	6	2927622	4.46E-10	*qDFF6.1*
	KNL20	AX182197986	8	2590145	4.65E-08	*qDFF8.2*
	MDP19	AX182223130	1	17102397	3.17E-12	*qDFF1.1*
	MDP19	AX182223369	5	29587984	6.22E-08	*qDFF5.1*
	MDP19	AX154607945	5	5977377	2.68E-07	*qDFF5.2*
	MDP19	AX154828491	6	2927622	2.68E-08	*qDFF6.1*
	MDP19	AX123164507	8	2208785	2.59E-07	*qDFF8.1*
	MDP20	AX182197986	8	2590145	1.20E-06	*qDFF8.2*
	RKR19	AX155029421	2	21916667	1.77E-08	*qDFF2.1*
	RKR19	AX154828491	6	2927622	3.09E-13	*qDFF6.1*
	RKR19	AX123164507	8	2208785	9.95E-12	*qDFF8.1*
	RKR19	AX182222307	8	11672536	4.65E-08	*qDFF8.3*
	RKR20	AX154828491	6	2927622	5.51E-11	*qDFF6.1*
	RKR20	AX117352818	11	27605483	5.88E-08	*qDFF11.1*
PHT	DEL20	AX154964191	1	38365392	2.23E-10	*qPHT1.1*
	DEL20	AX154680472	2	23979523	4.37E-07	*qPHT2.1*
	KNL19	AX154964191	1	38365392	6.22E-08	*qPHT1.1*
	KNL19	AX154811934	10	6148264	2.20E-09	*qPHT10.1*
	KNL19	AX154308002	12	1241440	5.15E-11	*qPHT12.1*
	KNL20	AX154499972	1	41157155	1.58E-07	*qPHT1.2*
	MDP19	AX155203878	1	38812075	1.34E-11	*qPHT1.3*
	MDP19	AX154499972	1	41157155	2.04E-07	*qPHT1.2*
	MDP19	AX154458544	5	29470794	1.41E-06	*qPHT5.1*
	MDP19	AX154308002	12	1241440	9.84E-21	*qPHT12.1*
	MDP20	AX154629125	2	23871370	1.60E-07	*qPHT2.2*
	MDP20	AX154720065	5	25531280	6.51E-07	*qPHT5.2*
	MDP20	AX154289720	6	4527143	1.34E-07	*qPHT6.1*
	RKR19	AX154964191	1	38365392	1.08E-10	*qPHT1.1*
	RKR19	AX154499972	1	41157155	5.61E-07	*qPHT1.2*
	RKR19	AX115863287	2	24149413	1.35E-14	*qPHT2.3*
	RKR20	AX154964191	1	38365392	9.87E-10	*qPHT1.1*
	RKR20	AX154732161	1	33882896	1.66E-07	*qPHT1.4*
	RKR20	AX154391823	12	6128687	6.56E-09	*qPHT12.2*
PL	DEL20	AX155128484	2	14989190	3.20E-07	*qPL2.1*
	DEL20	AX155902244	6	1181984	9.26E-09	*qPL6.1*
	DEL20	AX182309471	10	1307094	1.57E-06	*qPL10.1*
	KNL20	AX182209644	2	20099298	2.49E-07	*qPL2.2*
	KNL20	AX182198943	5	7982455	5.86E-08	*qPL5.1*
	KNL20	AX182222834	11	16342594	1.05E-11	*qPL11.1*
	MDP19	AX182202609	2	31595743	4.33E-08	*qPL2.4*
	MDP19	AX154944620	2	18464600	5.66E-08	*qPL2.5*
	MDP19	AX182211809	3	16216860	2.55E-09	*qPL3.2*
	MDP19	AX154004277	9	17887900	2.22E-10	*qPL9.1*
	MDP19	AX182222834	11	16342594	2.99E-07	*qPL11.1*
	MDP19	AX154581132	12	269451	1.59E-11	*qPL12.1*
	MDP20	AX154060362	3	30248393	1.39E-07	*qPL3.1*
	MDP20	AX182222834	11	16342594	7.53E-10	*qPL11.1*
	RKR19	AX182210242	2	25793464	3.56E-08	*qPL2.3*
	RKR19	AX182202609	2	31595743	3.38E-07	*qPL2.4*
	RKR20	AX155902244	6	1181984	4.44E-10	*qPL6.1*
	RKR20	AX182222834	11	16342594	1.28E-09	*qPL11.1*

MTA, Marker trait association; DFF, Days to fifty percent flowering; PHT, Plant height; PL, Panicle length.

**Figure 6:**
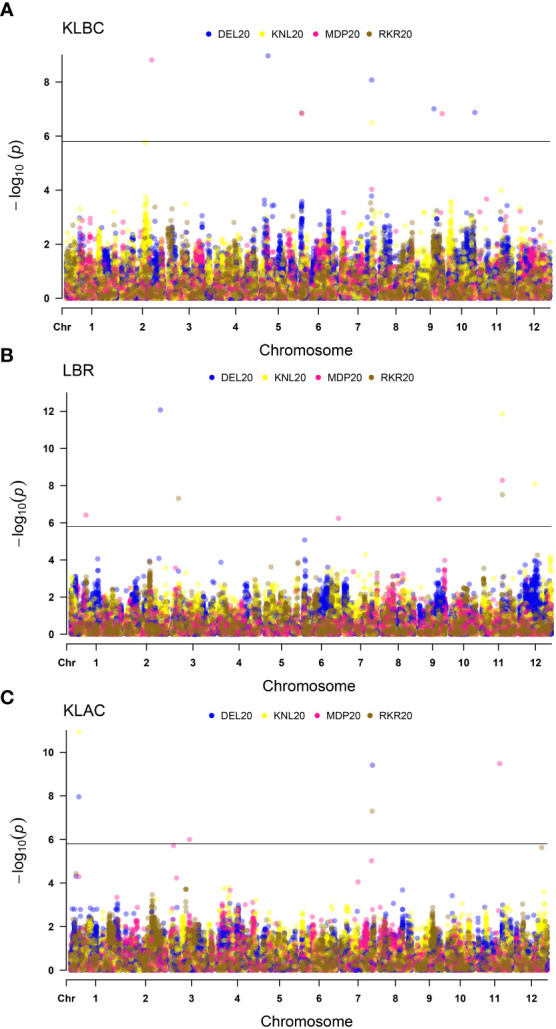
Multi-Environmental Manhattan plots depicting significant MTAs for various grain quality traits *viz*. **(A)** Kernel length before cooking (KLBC), **(B)** Length-Breadth Ratio (LBR) and **(C)** Kernel length after cooking (KLAC). The dots marked in different colours indicate QTNs detected in different environment. The horizontal line indicates Bonferoni cut-off.

For the grain quality parameters, the MTAs detected are summarized in **Table 4**. The Manhattan plots (**Figure 6**), indicated the presence of 29 MTAs, which were significant based on the threshold limit. Ten MTAs have been identified for KLBC, including nine unique QTNs. One QTN i.e., *qKLBC7.1* was identified in more than one location (DEL20 and KNL20). Similarly, nine significant MTAs were found for LBR of which five QTNs were unique in their occurrence across locations. The QTN, *qLBR11.1* identified in three environments was recognised as the most stable QTN for LBR. In the case of KLAC, 10 MTAs were detected and all the detected significant QTNs were identified only in individual environments. Among these, one QTN was each found on chromosomes 4, 10 and 12, while, two each were located on chromosomes 2 and 7 and there were three QTNs on chromosome 1.

**Table 4:** Marker trait associations (MTA) detected in GWAS by using the BLINK model for the grain quality traits in four environments.

Trait	Environment	SNP	Chromosome	Position	P value	MTA
KLBC	DELHI20	AX154262342	5	6161273	1.07E-09	*qKLBC5.1*
	DELHI20	AX154772156	7	26160944	8.41E-09	*qKLBC7.1*
	DELHI20	AX154137289	9	14559958	9.74E-08	*qKLBC9.1*
	DELHI20	AX115761775	10	22867856	1.33E-07	*qKLBC10.1*
	KNL20	AX154890753	2	20355507	1.69E-06	*qKLBC2.1*
	KNL20	AX154772156	7	26160944	3.26E-07	*qKLBC7.1*
	MDP20	AX154377556	2	25829978	1.53E-09	*qKLBC2.2*
	MDP20	AX182194057	6	1538424	1.48E-07	*qKLBC6.1*
	MDP20	AX154996118	9	21652502	1.48E-07	*qKLBC9.2*
	RKR20	AX153907646	6	1182044	1.37E-07	*qKLBC6.2*
LBR	DEL20	AX123152303	2	29869184	8.41E-13	*qLBR2.1*
	KNL20	AX182204490	11	17932922	1.37E-12	*qLBR11.1*
	KNL20	AX182308110	12	13349687	8.40E-09	*qLBR12.1*
	MDP20	AX154652825	1	12890642	3.85E-07	*qLBR2.1*
	MDP20	AX154339001	6	30363945	5.75E-07	*qLBR6.1*
	MDP20	AX153977028	9	16947546	5.28E-08	*qLBR9.1*
	MDP20	AX182204490	11	17932922	5.19E-09	*qLBR11.1*
	RKR20	AX155136144	3	5833059	4.81E-08	*qLBR3.1*
	RKR20	AX182204490	11	17932922	3.08E-08	*qLBR11.1*
KLAC	DELHI20	AX154262289	1	7134557	2.37E-11	*qKLAC1.1*
	DELHI20	AX115787778	7	26969400	2.81E-16	*qKLAC7.1*
	KNL20	AX153938644	1	4729873	2.92E-07	*qKLAC1.2*
	KNL20	AX182210295	2	26280744	2.23E-08	*qKLAC2.1*
	KNL20	AX154293128	2	23458209	1.99E-07	*qKLAC2.2*
	MDP20	AX155248360	1	4299523	1.65E-07	*qKLAC1.3*
	MDP20	AX182185736	4	17576441	1.07E-07	*qKLAC4.1*
	MDP20	AX154364235	7	28752139	8.41E-07	*qKLAC7.2*
	RKR20	AX154293360	10	19992776	3.16E-07	*qKLAC10.1*
	RKR20	AX182185892	12	6738828	2.70E-08	*qKLAC12.1*

MTA, Marker trait association; KLBC, Kernel length before cooking; LBR, Length-Breadth Ratio; KLAC, Kernel length after cooking.

### 3.4 Allelic effects and combinations of stable quantitative trait nucleotides

#### 3.4.1 Agronomic traits

For all the 13 stable MTAs identified across environments associated with agronomic and grain quality traits, allelic differences were large and significant based on simple linear regression. For DFF, the allele ‘C’ (90.89 days) of *qDFF6.1* hastened the flowering of genotypes by about 15 days as against its alternate allele ‘G’ (105.02 days) (**Supplementary Figure 1**). In the case of *qDFF8.1*, the genotypes carrying allele ‘G’, recorded an average DFF of 91.97 days, while that of its alternate allele ‘C’ had 105.53 DFF with an allelic advantage of 13.56 days. For *qDFF8.2*, the genotypes with the allele ‘T’ registered 87.47 days whereas those with allele ‘C’ showed 99.45 days with an allelic difference of 11.98 days. The MTA, *qDFF11.1*, though detected in only two environments exhibited the largest effect of 15.07 days. The allele ‘G’ of *qDFF11.1* had a mean trait value of 91.48 days and that of the ‘C’ allele was 106.55 days. The QTN, *qDFF5.1* registered the lowest allelic effect of 7.96 days with its alternate allele’s ‘C’ and ‘T’ having an average effect of 87 days and 94.96 days respectively (**Table 5**). Looking at the combinatorial effects of the QTNs, a total of 10 combinations were available (**Supplementary Figure 2**) among the genotypes in the panel. Subsequent comparison of their effect obtained by pooling the genotypic values from all seven environments revealed that the combination ‘TCCCT’ was found to confer the longest DFF (108.74 days), whereas the combination ‘CGTGC’ (87.5 days) had the shortest DFF. However, the predominant allelic combination was ‘CGTGT’ with an average DFF of 90.03, albeit, at par with the combination ‘CGTGC’. The combinations one to six showed DFF of above 100 days whereas the combinations from seven to 10 were below 93 days (**Table 6**).

**Table 5:** Allelic effects of stable MTAs identified in multiple locations for Days to fifty Per cent flowering (DFF).

SNP ID	QTN	Ch	Pos	Previous report	FA	AA	Environment	Trait mean		P value
								FA	AA	
AX154828491	*qDFF6.1*	6	2927622	*RFT1* (Komiya et al., 2009)	C	T	DEL20	92.24	101.04	1.17E-06
							KNL19	93.07	107.12	4.06E-21
							KNL20	87.47	106.46	9.3E-22
							MDP19	92.57	102.83	2.24E-20
							RKR19	90.62	106.14	5.59E-22
							RKR20	89.38	106.53	1.13E-23
							**Grand Mean**	**90.89**	**105.02**	
AX123164507	*qDFF8.1*	8	2208785	*Hd18* (Shibaya et al., 2016)	G	C	KNL19	92.91	107.54	6.48E-25
							MDP19	92.43	103.19	2E-24
							RKR19	90.57	105.85	2.61E-23
							**Grand Mean**	**91.97**	**105.53**	
AX182197986	*qDFF8.2*	8	2590145	*Hd18* (Shibaya et al., 2016)	T	C	KNL20	87.38	101.58	3.33E-15
							MDP20	87.55	97.31	8.09E-17
							**Grand Mean**	**87.47**	**99.45**	
AX117352818	*qDFF11.1*	11	27605483	–	G	C	KNL19	93.01	107.96	9.85E-24
							RKR20	89.95	105.13	1.14E-16
							**Grand Mean**	**91.48**	**106.55**	
AX182223369	*qDFF5.1*	5	29587984	–	C	T	DEL20	84.78	94.92	0.00131
							MDP19	89.22	95.00	0.005033
							**Grand Mean**	**87.00**	**94.96**	

SNP, Single nucleotide polymorphism; QTN, Quantitative trait nucleotide; Ch, Chromosome; Pos, Position; FA, Favourable allele; AA, Alternate allele.Bold values presented in this table indicates the 'Grand Mean' under individual QTNs.

**Table 6:** List of all available homozygous allelic combinations of selected QTNs for DFF.

Combination number	*qDFF6.1*	*qDFF8.1*	*qDFF8.2*	*qDFF11.1*	*qDFF5.1*	Pooled DFF^#^	No. of genotypes
1	T	C	C	C	T	108.74^a^	16
2	T	C	C	G	T	107.96 ^a^	2
3	T	G	T	C	T	106.09 ^ab^	5
4	C	C	C	C	T	102.41 ^b^	6
5	C	C	C	G	T	102.11 ^b^	9
6	T	G	T	G	T	101.08 ^b^	6
7	C	G	C	G	T	92.17 ^c^	10
8	C	G	T	C	T	92.15 ^c^	4
9	C	G	T	G	T	90.03 ^c^	89
10	C	G	T	G	C	87.5 ^c^	9

^#^The values with same letters as superscripts are at par at p<0.05.

For PHT, the allele ‘A’ of *qPHT1.1* displayed an average plant height of 104.36 cm, and the effect of allele ‘G’ was estimated at 121.76 cm, with a difference of 17.4 cm. Similarly, the genotypes with allele ‘T’ of *qPHT1.2* showed a height of 105.37 cm, while those with allele ‘C’ registered 122.86 cm. Thus, the allelic contribution of *qPHT1.2* was 17.49 cm. The allele ‘T’ of the third MTA, *qPHT12.1* registered an average plant height of 87.92 cm and allele ‘C’ showed 101.87 cm (**Supplementary Figure 3**), the difference between these alleles being 13.95 cm (**Table 7**). Allelic combinations of three stable MTAs of PHT revealed five unique allelic combinations (**Supplementary Figure 4**). The combination ‘ATT’ was found to be predominant (69.1%) in the population with an average plant height of 107.74 cm. Of the remaining, ‘GCT’ registered the highest plant height of 129.8 cm while the lowest plant height (95.18 cm) was obtained for the combination ‘ATC’. However, GCT was found among 2.9%, while ATC was seen only among 5.8% of the population. The remaining genotypes were heterozygous allelic mixtures at these target loci (**Table 8**).

**Table 7:** Allelic effects of selected MTAs identified in multiple locations for plant height (PHT).

SNP ID	QTN	Ch	Pos	Previous report	FA	AA	Environment	Trait mean (cm)		P value
								FA	AA	
AX154964191	*qPHT1.1*	1	38365392	*SD1* (Sasaki et al., 2002)	A	G	DEL20	109.13	135.09	4.47E-21
							KNL19	97.66	109.13	5.26E-14
							RKR19	101.45	114.74	2.67E-10
							RKR20	109.20	128.07	1.81E-13
							**Grand mean**	**104.36**	**121.76**	
AX154499972	*qPHT1.2*	1	41157155	*qPHT1-1* (Hittalmani et al., 2003)	T	C	KNL20	113.27	129.27	4.15E-07
							MDP19	100.77	118.71	8.13E-09
							RKR19	102.06	120.61	4.81E-09
							**Grand mean**	**105.37**	**122.86**	
AX154308002	*qPHT12.1*	12	1241440	–	T	C	KNL19	89.52	100.30	5.42E-06
							MDP19	86.32	103.44	3.17E-08
							**Grand mean**	**87.92**	**101.87**	

SNP, Single nucleotide polymorphism; QTN, Quantitative trait nucleotide; Ch, Chromosome; Pos, Position; FA, Favourable allele; AA, Alternate allele.Bold values presented in this table indicates the 'Grand Mean' under individual QTNs.

**Table 8:** List of all available homozygous allelic combinations of selected QTNs for PH.

Combination number	*qPHT1.1*	*qPHT1.2*	*qPHT12.1*	Pooled PHT (cm)^#^	No. of genotypes
1	G	C	T	129.98 ^a^	5
2	G	T	T	120.38 ^ab^	18
3	A	C	T	108.94 ^b^	4
4	A	T	T	107.74 ^b^	119
5	A	T	C	95.18 ^c^	10

^#^The values with same letters as superscripts are at par at p <0.05.

Of the three stable MTAs for PL, alleles of the MTA, *qPL6.1* were found to confer the PL difference of 2.68 cm (**Table 9**). The allele ‘G’, showed an average PL of 28.43 cm whereas the allele ‘A’ registered 25.75 cm. The MTA, *qPL2.4*, has shown an effect of 1.56 cm with its alleles, ‘T’ and ‘G’ showing a PL of 26.73 cm and 28.29 cm, respectively. The MTA, *qPL11.1* was found to be the most stable MTA as it was detected consistently across four environments. The allele ‘T’ of *qPL11.1* had an average panicle length of 28.76 cm, while allele ‘C’ attributed a panicle length of 27.36 cm. The distribution for PL for these MTAs is depicted in, **Supplementary Figure 5**. Six unique homozygous allelic combinations were identified among the association panel for PL-related MTAs (**Table 10**). The allelic combination 1, TGT, displayed the highest panicle length of 29.31 cm, while combination 6 (GAC) was identified with the lowest panicle length (24.7 cm). The combination, TGC, was found to be the most frequent combination in the panel with a mean panicle length of 28.02 cm (**Supplementary Figure 6**).

**Table 9:** Allelic effects of selected MTAs identified in multiple locations for Panicle Length (PL).

SNP ID	QTN	Ch	Pos	Previous report	FA	AA	Environments	Trait mean (cm)		P value
								FA	AA
AX182202609	*qPL2.4*	2	31595743	*OsSK1* (Kasai et al., 2005)	T	G	RKR19	28.39	26.67	3.6655E-09
							MDP19	28.19	26.79	4.48631E-10
							**Grand mean**	**28.29**	**26.73**	
AX155902244	*qPL6.1*	6	1181984	–	G	A	DEL20	29.05	26.39	1.0525E-05
							RKR20	27.81	25.11	1.13679E-06
							**Grand mean**	**28.43**	**25.75**	
AX182222834	*qPL11.1*	11	16342594	–	T	C	KNL20	28.71	27.20	4.34108E-09
							RKR20	28.23	26.47	6.12007E-11
							MDP20	29.81	28.07	1.27599E-09
							MDP19	28.31	27.70	0.00602236
							**Grand mean**	**28.76**	**27.36**	

SNP, Single nucleotide polymorphism; QTN, Quantitative trait nucleotide; Ch, Chromosome; Pos, Position; FA, Favourable allele; AA, Alternate allele.Bold values presented in this table indicates the 'Grand Mean' under individual QTNs.

**Table 10:** List of all available homozygous allelic combinations of selected QTNs for PL.

Combination number	*qPL2.4*	*qPL6.1*	*qPL11.1*	Pooled PL (cm)^#^	No. of genotypes
1	T	G	T	29.31 ^a^	23
2	G	G	T	28.15 ^ab^	18
3	T	G	C	28.02 ^ab^	92
4	T	A	C	26.7 ^b^	7
5	G	G	C	26.64 ^b^	16
6	G	A	C	24.7 ^c^	1

^#^The values with same letters as superscripts are at par at p<0.05

#### 3.4.2 Grain quality traits

The allelic effect of the two stable MTAs identified for two grain quality traits, KLBC and LBR indicated that (**Supplementary Figure 7** and **Table 11**), the allele A of *qKLBC7.1* has an average kernel length of 8.07 mm and its alternate allele ‘G’ has shown a kernel length of 7.16 mm. Their effect due to allelic difference was estimated to be 0.91 mm and found to be highly significant across all detected environments. For LBR, the favourable allele ‘T’ of the MTA *qLBR11.1* has shown an LBR of 4.81 and its alternate allele ‘C’ is estimated at 4.1, with a significant allele difference of 0.71.

**Table 11:** Allelic effects of selected MTAs identified in multiple locations for the grain quality traits trait, KLBC and LBR.

Trait	SNP ID	QTN	Ch	Pos	Previous report	FA	AA	Environments	Trait mean		P value
									FA	AA	
KLBC	AX154772156	*qKLBC7.1*	7	26160944	*qGL7.2* (Shao et al., 2010)	A	G	DELHI20 (mm)	8.01	7.08	6.57E-12
								KNL20 (mm)	8.13	7.24	8.51E-13
								**Grand mean (mm)**	**8.07**	**7.16**	
LBR	AX182204490	*qLBR11.1*	11	17932922	*-*	T	C	KNL20	4.93	4.21	4.14E-07
								MDP19	4.92	4.10	3.14E-09
								RKR20	4.57	3.99	7.18E-07
								**Grand mean**	**4.81**	**4.1**	

KLBC, Kernel length before cooking; LBR, Length-Breadth Ratio; QTN, Quantitative trait nucleotide; Ch, Chromosome; Pos, Position;FA, Favourable allele; AA, Alternate allele.Bold values presented in this table indicates the 'Grand Mean' under individual QTNs.

### 3.5 Selection of genotypes with superior allelic combinations

**Figure 7:**
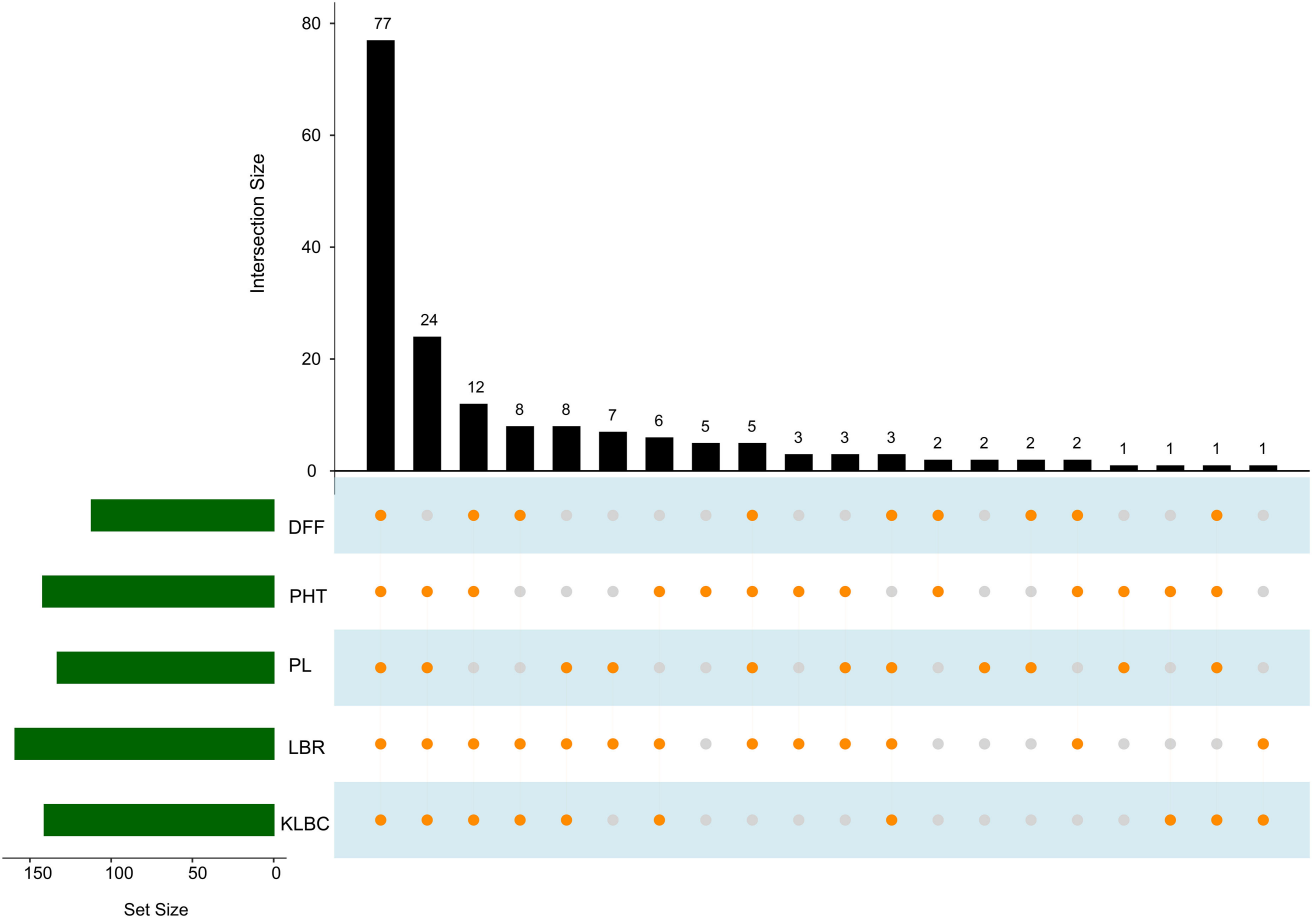
Upset plot indicating genotype groups for elite allelic combinations for each trait. Green set size bar indicating the number of selected genotypes for each trait. Orange dots connected by lines corresponding to the bar graph indicate the number of selected genotypes which overlaps among traits. (DFF, Days to fifty percent flowering; PHT, Plant height in cm; PL, Panicle length in cm).

Genotype selection targeting short to medium growth duration, using the allelic combinations 7 to 10 identified 112 genotypes (**Table 6**). Following the ideal agronomic preference, the semi-dwarf to semi-tall genotypes are preferred for restorers. Using the allele combinations 1 to 4 of the MTAs associated with PHT, 105 putative restorers were identified (**Table 8**). Similarly, semi-dwarf plant types are favoured as maintainers, and hence using allele combinations 3 to 5, 37 putative maintainers were selected. Similarly, allele combinations 1 to 3 were ideal for PL, resulting in the selection of 133 genotypes having longer panicle lengths (**Table 10**). Selection for Basmati grain quality was based on two traits, KLBC and LBR. A higher value of KLBC is ideal for Basmati genotypes, therefore, the ‘A’ allele of the stable MTA, *qKLBC7.1* was preferred and 141 individuals were carrying this particular allele. Similarly, the ‘T’ allele of *qLBR11.1* was associated with a higher LBR value; accordingly, 159 genotypes were chosen. Finally, the upset plot revealed that 77 genotypes carry all desirable allelic combinations for the five traits (**Figure 7**) the details of which are provided in **Supplementary Table 6**.

## 4 Discussion

GWAS uses statistical prediction on the loci significantly influencing the traits (MTAs) through iterative procedures built around various regression models. This is particularly important with complex traits governed by a multitude of genomic regions, where the expression can be largely influenced by allelic effects, interactions as well as environments. Further, GWAS uses filters such as population structure and kinship (co-ancestry) to nullify spurious associations. Mapping of MTAs through GWAS also permits the identification of favourable allelic combinations vis-à-vis trait expression. This is critical for a breeding programme aimed to integrate favourable alleles during crop improvement (Tibbs Cortes et al., 2021). Such an exercise is equally important in Basmati rice improvement, where balanced selection for various traits is decisive in determining critical parental combinations for hybrid breeding. In the present study, we have used this approach on a diverse but elite multiparent breeding population of Basmati rice derived from set of Basmati and non-Basmati founder parents. The non-Basmati founders were utilized for targeting the agronomic improvement, while the Basmati founders contributed to the grain and cooking quality traits in the improved elite lines. To our knowledge, this is the first attempt in a Basmati rice breeding population. The association mapping panel with 172 lines showed significant phenotypic variation for agronomic traits coupled with key Basmati traits such as aroma and long slender grains. Out of these, however, only 141 of these lines could qualify as Basmati based on the minimum standards for all the traits defined for Basmati classification in India (Singh et al., 2018). Having further examined under multi-environmental trials for agronomic and grain quality attributes, based on rank correlations, the environments proved to provide comparable influence on the genotypes irrespective of the magnitude. Similar ranking of the lines across environments indicated lesser crossover genotype by environment interaction, which renders them ideal for GWAS. This information is highly relevant in this context because the four locations used, Delhi, Rakra, Karnal and Modipuram represent four administrative states within the Basmati GI region in Northern India, *viz*., Delhi, Punjab, Haryana, and Western Uttar Pradesh. Moreover, the phenotypic variation among the lines was highly encouraging across all the locations, except for traits such as PL. The phenotypic evaluation also hinted a consistent and stark difference in PHT and DFF over the same locations, between *Kharif* 2019 and *Kharif* 2020, resultant of difference in the sowing dates: entries were sown late in *Kharif* 2019 (first week of July), while timely sowing was adopted in *Kharif* 2020 (first week of June). The effect of sowing and transplanting time on the agronomic performance of aromatic rice, especially in Basmati types is well documented (Bhattacharjee et al., 2002; Singh et al., 2013).

The panel showed ample genetic variation, with 52.8% genome-wide divergence. Furthermore, the study is unique in several ways – it is one of the first studies that utilized the 80K SNP array as well as an improved multiparent population composed of elite Basmati breeding lines as an association mapping panel. The population structure analysis revealed around 45% of admixtures, which is expected due to fewer recombination events during population development from the founder parents with one cycle as compared to germplasm which accumulates a large number of recombinations due to long history of evolution. Furthermore, the Basmati lineage in the rice gene pool has a restricted genetic variability, when compared to other cultivated rice lineages, which also can lead to larger haplotypes within the population. This was apparent from the LD decay within the panel which approximately elbowed at 1.8Mb, the value ordinarily larger than the LD decay reported in rice germplasms (Zhao et al., 2011; Huang et al., 2012). Besides, the number of recombination events in the current panel is low, because the population has just undergone six years of breeding activity leading to slow LD decay (Ponce et al., 2020). Thus, the LD decay pattern in our population mirrors the breeding history and recent origin of the breeding population.

GWAS using germplasm and the heterogenetic stock has been attempted for grain quality traits including grain dimensions (Yang et al., 2019; Zaw et al., 2019; Ponce et al., 2020; Zhong et al., 2021) and grain appearance (Qiu et al., 2021; Misra et al., 2019; Zaw et al., 2019; Zhong et al., 2021). To our knowledge, there are limited GWAS studies on grain quality traits of rice, particularly on cooking quality. In a recent study, Cruz et al. (2021) used a set of 284 multi-parent derived rice breeding lines for GWAS and genomic selection of grain quality traits. The traits investigated in the present study were crucial for the breeding programs aiming for the development of parental lines for hybrid development and improvement of grain quality in Basmati rice. Synchronization of flowering is one of an important consideration which determines better nicking of parents during hybrid seed production (Gaballah et al., 2021). The MTA, *qDFF6.1* was detected as the most reliable QTN for DFF, since its presence was evident in most of the locations and lies within the *RFT1* gene (*Os06g0157500*), which is directly involved in controlling flowering time in rice (Komiya et al., 2009). This QTN hence can be designed as a functional marker for the selection of DFF. Two stable MTAs, *qDFF8.1* and *qDFF8.2* were also found flanking upstream and downstream of the gene *Hd18* (*Os08g0143400*) that governs flowering time in rice (Shibaya et al., 2016). *Hd18* was located 176.7Kb downstream of the MTA, *qDFF8.1* and 200.8Kb upstream of another MTA, *qDFF8.2. RFT1* gene promotes flowering in short-day conditions, whereas *Hd18* promotes flowering in both short-day and long-day conditions (Li et al., 2018). No previously reported QTLs were found in the vicinity of two MTAs, *qDFF11.1* and *qDFF5.1*, hence these can be considered novel. Thus, these MTAs could be valuable candidates for selection of flowering behaviour in the population. This is further supported by the distinct effect of allelic combinations on flowering signifying that these three MTAs play a key role in deciding the flowering time and hence can be efficiently used in marker assisted selection.

Plant height is another important factor in hybrid seed production as optimum plant height combination in the male and female parents can facilitate better pollen flow (Mao et al., 1998). The MTA *qPHT1.1* identified as the most stable MTA was found to colocalize with the *sd1* (*Os01g0883800*), the green revolution gene (Spielmeyer et al., 2002). *qPHT1.1* was located 17Kb upstream of the *SD1* locus (Sasaki et al., 2002). The allelic combination study revealed that *qPHT1.1* was the most critical in deciding the plant height in the population compared to other two MTAs. The MTA, *qPHT1.2* was found to colocalize with a previously reported QTL for plant height, *qPHT1-1* (Hittalmani et al., 2003). However, *qPHT12.1* is novel, since no relevant reports were found for the associated genomic region. It is particularly interesting to note that *qPHT12.1* was found to influence height reduction further, even when present along with semidwarf variants of the other two MTAs. However, its individual effect could not be deciphered from the current panel, due to the lack of suitable allelic combinations at all the loci. The length of the panicle is yet another primary trait positively associated with grain yield and specifically desired among the rice hybrids (Kulkarni et al., 2020). Out of the three stable MTAs for panicle length, *qPL2.4* was found to be located downstream of a gene, *OsSK1* (*Os02g0749300*), which plays a significant role in panicle development (Kasai et al., 2005). The role of *OsSK1* in the production of shikimate 3-phosphate and thereby influencing panicle development is well illustrated by Kasai et al. (2005). We could not find any potential candidate genes/QTLs, near the remaining MTAs (*qPL6.1* and *qPL11.1*), and therefore, are considered unreported. The allelic combination study indicated that all three MTAs were having a minor but cumulative effect on panicle length, hence selection for the best allelic combination of the three MTAs can reward higher panicle length.

As mentioned earlier, maintenance of grain quality is pivotal to Basmati rice improvement, as the acceptability for Basmati is associated with grain parameters. Since the current panel was developed from diverse parental lines including elite Basmati varieties, we did not expect a significant diversity for grain parameters. Contrary to this, the entire panel exhibited excellent grain quality mostly conforming to Basmati standards. Yet, the association study revealed one stable MTA each for KLBC and LBR. No stable MTAs were found for KLAC reflecting the sensitivity of this trait to the environment (Rajendran et al., 2021). The MTA for KLBC, *qKLBC7.1* was found to co-localize with the QTL for grain length*, qGL7.2* **(** Shao et al., 2010). However, the MTA identified for LBR, *qLBR11.1*, is novel for this report, as no known associated genomic regions are found flanking this MTA. This proves that despite a careful and targeted selection for Basmati grain quality standards, at the various stages of generation advancement, the panel could still throw out major alleles governing elite grain quality. Additionally, the detected MTAs indicate the existence of latent variability for key Basmati traits in the elite breeding pool.

Finally, the comparison of genotypes carrying positive allelic combinations for agronomic and grain quality traits within the panel revealed 77 superior genotypes, this includes 72 putative restorers and 5 maintainers. The selected lines roughly covered 45% of the panel. We presume that these promising lines can be potential candidates for intercrossing to generate populations for the next selection cycle or also can be directly used as parental lines for the hybrid breeding programme, which is being investigated in a separate study. The MTAs identified in the present study can help in the fixation of valuable alleles in the subsequent cycles of improvement through precise marker-assisted selection while maintaining genetic diversity. This would not only be helpful in enhancing heterosis by identifying diverse parental lines but also enable further improvement in subsequent cycles without compromising on the genetic diversity. Also, we foresee that the data from recombinations from subsequent breeding cycles will help us to delineate the haplotypes which can be utilized in precise improvement in the future. Besides, the remaining genotypes can also be tested for their potential in hybrid breeding towards maximising heterosis. Further, this study can serve as a model for generating superior parents through systematic intermating among restorers and maintainers founders for hybrid breeding in other crops as well. A genomic selection approach using this panel could open opportunities for synthesizing and predicting several hybrid combinations, eliminating the need for attempting a large number of random test crosses for identifying heterotic Basmati hybrids.

## 5 Conclusion

In the present study, we performed a GWAS for agronomic and grain quality traits on a synthesised parental pool of Basmati rice to explore the accumulation of favourable candidate genomic regions and their allelic combinations. Several stable MTAs, particularly those proximal to already reported genes, served as valuable candidates to select superior genotypes from the panel. Among the 57 MTAs identified for various traits, only 22.8% were found stable implying a significant influence of environments on trait expression. These results suggested that the population improvement carried out for the development of a Basmati parental pool, in which the multiple founder parent hybridization was undertaken and several desired loci have been successfully accumulated. This provides an impetus for using these elite lines for superior Basmati rice hybrid development. Interestingly, the identification of MTAs for grain quality attributes further elevates the utility of the improved panel, aimed toward the improvement of grain quality among the potential future Basmati hybrids.

## Data availability statement

The authors acknowledge that the data presented in this study are deposited in the KRISHI Publication and Data Inventory Repository, available at https://krishi.icar.gov.in/jspui/handle/123456789/73842.

## Author contributions

SGK conceived the idea and formulated the research plan. KPA carried out the research work and prepared the manuscript. SGK, GD and PK generated the elite breeding lines. PKB, MN, RaS, RiS and SKB assisted in the execution of field trials. KKV, RKE, HB and KTR assisted in data analysis. SGK and KKV improved the manuscript. SGK and AKS provided overall guidance for the study. This work forms a part of the doctoral research work of KPA. All authors have read the article and approved the final version.

## Funding

This research was funded by the National Agricultural Higher Education Project (NAHEP) – Centre for Advanced Agricultural Science and Technology (CAAST) project (Code: 12-115) on “Genomics Assisted Breeding for Crop Improvement,” of the World Bank and the Indian Council of Agricultural Research, New Delhi.

## Conflict of interest

The authors declare that the research was conducted in the absence of any commercial or financial relationships that could be construed as a potential conflict of interest.

## Publisher’s note

All claims expressed in this article are solely those of the authors and do not necessarily represent those of their affiliated organizations, or those of the publisher, the editors and the reviewers. Any product that may be evaluated in this article, or claim that may be made by its manufacturer, is not guaranteed or endorsed by the publisher.
