# Supplementary material for: Genome-wide association study reveals novel genomic regions governing agronomic and grain quality traits and superior allelic combinations for Basmati rice improvement
Source: Front Plant Sci. 2022 Dec 5;13:994447. doi: 10.3389/fpls.2022.994447 (PMC9760805; doi:10.3389/fpls.2022.994447)

Genome-wide association study reveals novel genomic regions governing agronomic and grain quality traits and superior allelic combinations for Basmati rice improvement

Krishnan P. Abhijith, S. Gopala Krishnan, Kuram Tirumala Ravikiran, Gaurav Dhawan, Pankaj Kumar, Kunnummal Kurungara Vinod, Prolay Kumar Bhowmick, Mariappan Nagarajan, Rakesh Seth, Ritesh Sharma, Sourav Kumar Badhran, Haritha Bollinedi, Ranjith Kumar Ellur and Ashok Kumar Singh

Front. Plant Sci., Sec. Technical Advances in Plant Science
doi: 10.3389/fpls.2022.994447

Supplementary information

Supplementary Table 1: List of the founder parents used for generating elite rice breeding line panel

Founder Parents	Type	DFF (Days)	Plant height Class	Grain type	Aroma
PB 1121	Basmati	112	Semi dwarf	Extra-long slender	Aromatic
PB 1509	Basmati	85	Semi dwarf	Extra Long slender	Aromatic
PB 1609	Basmati	90	Semi dwarf	Long slender	Aromatic
PRR78	Basmati	90	Semi dwarf	Long slender	Aromatic
Punjab Basmati 3	Basmati	109	Semi dwarf	Long slender	Aromatic
Pusa 1401	Basmati	110	Semi dwarf	Long slender	Aromatic
Pusa 1568	Basmati	100	Tall	Extra-long slender	Aromatic
Pusa 1601	Basmati	92	Semi dwarf	Long slender	Aromatic
Pusa 1608	Basmati	105	Semi dwarf	Long slender	Aromatic
Pusa 1790	Basmati	92	Semi dwarf	Long slender	Aromatic
Pusa 1826	Basmati	110	Semi dwarf	Extra-long slender	Aromatic
Pusa 21B	Basmati	85	Semi dwarf	Long slender	Aromatic
Pusa 25B	Basmati	86	Semi dwarf	Long slender	Aromatic
Pusa 6B	Basmati	85	Semi dwarf	Long slender	Aromatic
AR46	Basmati	90	Semi dwarf	Long slender	Aromatic
SGW223	Non-Basmati	89	Semi dwarf	Long slender	Aromatic
ANP406	Non-Basmati	71	Tall	Short Slender	Aromatic
ANP416	Non-Basmati	90	Semi Tall	Short Slender	Aromatic
PRR50012	Non-Basmati	90	Semi tall	Long slender	Aromatic
PRR50019	Non-Basmati	89	Semi tall	Long slender	Aromatic
IET12014	Non-Basmati	87	Semi dwarf	Long slender	Aromatic
IET18033	Non-Basmati	75	Tall	Long slender	Aromatic
JGL11609	Non-Basmati	120	Semi dwarf	Short Slender	Aromatic
PKV Makarkand	Non-Basmati	91	Semi dwarf	Medium slender	Aromatic

Semi dwarf: 70-110 cm; Semi tall 110-130cm; Tall: >130cm; Extralong slender: Length ≥ 9mm and L/B ≥ 4.0; Long slender: Length ≥ 6mm and L/B ≥ 3.0; Medium slender: Length <6mm and L/B 2.5 to 3.0; Short Slender: Length < 6mm and L/B ≥ 3.0

Supplementary Table 2: ANOVA table showing genotypic mean square values of the traits in multiple environments

Environment	df	DFF	PHT	PL	KLBC	KLAC	LBR
DEL20	171	88.79**	194.87**	5.47**	0.5**	2.53**	0.27**
KNL20	171	176.86**	121.49**	4.87*	0.58**	3.12**	0.28**
MDP20	171	74.58**	125.88**	6.57**	0.49**	1.99**	0.22**
RKR20	171	138.15**	135.38**	3.95**	0.38**	2.34**	0.16**
KNL19	171	94.17**	67.8**	4.92*	NA	NA	NA
MDP19	171	39.03**	119.15**	4.25*	NA	NA	NA
RKR19	171	82.02**	135.53**	6.54**	NA	NA	NA

Single (*) asterisk denotes correlation is significant at 5%, whereas double (**) denotes correlation is significant at 1%, NA traits were not phenotyped in corresponding environments. DFF, Days to fifty percent flowering; PHT, Plant height in cm; PL, Panicle length in cm; KLBC, Kernel length before cooking in mm; LBR, Length-Breadth Ratio; KLAC, Kernel length after cooking in mm.

Supplementary Table 3: Tables showing Spearman's rank correlation between the environments for agronomic and grain quality traits. Environments are coded as KNL19: Karnal 2019; KNL20: Karnal 2020; DEL20: Delhi 2020; MDP19: Modipuram 2019; MDP20: Modipuram 2020; RKR19: Rakhra 2019; RKR20: Rakhra 2020

A. Days to 50% flowering

	KNL20	MDP20	DEL20	RKR20	KNL19	MDP19	RKR19
KNL20	1.00						
MDP20	0.86	1.00					
DEL20	0.82	0.89	1.00				
RKR20	0.71	0.70	0.66	1.00			
KNL19	0.70	0.74	0.73	0.59	1.00		
MDP19	0.70	0.72	0.69	0.61	0.80	1.00	
RKR19	0.67	0.74	0.75	0.60	0.75	0.79	1.00

B. Plant height (cm)

	RKR20	KNL20	KNL19	MDP19	RKR19	DEL20	MDP20
RKR20	1.00						
KNL20	0.46	1.00					
KNL19	0.59	0.55	1.00				
MDP19	0.53	0.55	0.80	1.00			
RKR19	0.46	0.48	0.79	0.74	1.00		
DEL20	0.71	0.57	0.61	0.54	0.51	1.00	
MDP20	0.65	0.60	0.65	0.58	0.48	0.77	1.00

C. Panicle length (cm)

PL	DEL20	RKR20	KNL20	MDP19	KNL19	MDP20	RKR19
DEL20	1.00						
RKR20	0.78	1.00					
KNL20	0.76	0.81	1.00				
MDP19	0.36	0.45	0.38	1.00			
KNL19	0.35	0.43	0.40	0.90	1.00		
MDP20	0.73	0.73	0.74	0.40	0.41	1.00	
RKR19	0.28	0.37	0.33	0.88	0.88	0.34	1.00

D. Kernel length before cooking (mm)

KLBC	DEL20	KNL20	RKR20	MDP
DEL20	1.00			
KNL20	0.66	1.00		
RKR20	0.65	0.65	1.00	
MDP	0.76	0.65	0.69	1.00

E. Kernel length after cooking (mm)

KLAC	DEL20	KNL20	RKR20	MDP
DEL20	1.00			
KNL20	0.77	1.00		
RKR20	0.73	0.72	1.00	
MDP	0.72	0.72	0.69	1.00

F. Length-width ratio

LWR	DEL20	KNL20	RKR20	MDP
DEL20	1.00			
KNL20	0.62	1.00		
RKR20	0.45	0.50	1.00	
MDP	0.45	0.49	0.49	1.00

Supplementary Table 4: BLUE values of Plant height (PHT), days to 50% flowering (DFF) and Panicle length (PL) of 172 breeding lines over 7 environments

Genotype	Location	*Kharif* 2020			*Kharif* 2019		
		PHT (cm)	DFF	PL (cm)	PHT (cm)	DFF	PL (cm)
GPR3	RKR	113.16	88.09	27.51	101.31	90.87	27.17
GPR4	RKR	111.81	88.05	28.96	97.86	88.91	27.67
GPR7	RKR	144.46	86.82	30.12	129.19	82.87	28.95
GPR8	RKR	120.91	82.17	28.27	123.76	84.91	28.4
GPR19	RKR	105.06	95.03	29	102.45	88.87	27.95
GPR21	RKR	112.99	90.89	27.71	98.18	100.87	26.03
GPR23	RKR	116.66	81.14	29.3	105.53	84.87	28.41
GPR24	RKR	109.88	90.21	28.76	106.81	88.87	29.34
GPR32	RKR	97.65	86.1	24.78	94.11	82.87	27.1
GPR35	RKR	103.03	83	27.34	98.52	81.2	28.93
GPR37	RKR	105.55	82.09	28.42	101.57	85.2	30.4
GPR38	RKR	112.95	90.16	27.24	100.82	90.87	27.62
GPR39	RKR	112.81	91.14	28.97	99.95	88.91	27.03
GPR42	RKR	110.48	92	27.73	NA	88.87	28.85
GPR43	RKR	93.57	83.02	21.82	130.82	90.87	26.87
GPR45	RKR	112.6	82.23	26.19	103.1	90.87	27.58
GPR47	RKR	158.25	84.04	26.56	100.7	90.87	26.52
GPR52	RKR	100.81	91.83	23.18	104.71	83.87	27.39
GPR60	RKR	103.46	108.85	28.83	99.28	104.91	26.39
GPR62	RKR	112.92	108.83	27.93	94.17	NA	26.94
GPR67	RKR	113.06	87.98	27.14	98.93	84.87	29.01
GPR70	RKR	112.72	85.09	26.88	101.79	86.91	28.63
GPR74	RKR	108.48	84.21	27.65	107.84	90.87	29.23

Genotype	Location	*Kharif* 2020			*Kharif* 2019		
		**PHT (cm)**	**DFF**	**PL (cm)**	**PHT (cm)**	**DFF**	**PL (cm)**
GPR77	RKR	110.15	94.7	27.81	101.64	88.91	26.84
GPR78	RKR	110.79	91.82	26.7	105.76	90.87	28.35
GPR80	RKR	109.96	90.78	27.4	108.36	87.91	28.43
GPR82	RKR	111.21	89.87	28.1	99.68	90.87	27.11
GPR86	RKR	98.78	89.84	25.09	102.62	89.2	28.73
GPR87	RKR	108.63	88.01	27.49	97.78	82.91	27.36
GPR92	RKR	111.3	88.04	27.84	104.08	88.87	28.26
GPR96	RKR	122.2	81.37	29.44	102.78	90.87	27.61
GPR100	RKR	117.51	81.38	28	106.9	88.87	29.26
GPR102	RKR	119.4	82.34	29.21	104.44	90.87	28.31
GPR104	RKR	121.19	84.22	27.94	104.19	91.2	27.31
GPR106	RKR	120.23	84.26	28.38	104.64	87.91	28.58
GPR111	RKR	104.79	92.2	27.95	86.38	91.2	25.33
GPR112	RKR	96.7	82.01	25.34	88.28	82.91	26.43
GPR113	RKR	105.93	90.31	27.17	87.74	94.87	26.41
GPR114	RKR	93.74	85.9	25.68	87.2	82.87	25.01
GPR115	RKR	109.2	90.84	26.32	103.42	90.87	27.31
GPR117	RKR	101.46	97.99	27.06	94.98	97.87	27.04
GPR118	RKR	101.99	96.06	27.3	97.79	93.2	27.68
GPR119	RKR	105.21	92.15	27.56	99.82	88.87	26.48
GPR120	RKR	98.78	89.84	25.09	97.4	91.2	27.91
GPR131	RKR	105.35	111.63	28.57	100.88	106.87	28.68
GPR136	RKR	112.35	88.73	28.57	93.64	106.91	28.54
GPR142	RKR	112.32	89.91	27.15	99.17	95.87	26.54
GPR144	RKR	112.94	88	26.95	105.5	90.87	27.3
GPR146	RKR	108.63	88.01	27.49	102.22	91.2	27.33
GPR147	RKR	107.42	91.76	28.35	104.66	94.87	28.12
GPR148	RKR	110.97	95.62	28.42	105.85	86.87	27.81
GPR149	RKR	109.2	89.96	27.66	101.27	91.2	28.23
GPR150	RKR	109.25	88.98	27.78	103.44	90.91	29.23
GPR151	RKR	120.21	91.86	27.19	89.04	88.87	27.05
GPR155	RKR	103.59	85.18	25.92	97.84	84.87	26.56
GPR157	RKR	111.8	85.15	24.53	96.34	82.87	25.56
GPR160	RKR	103.95	87.18	24.43	96.06	82.91	26.45
GPR164	RKR	107.78	82.13	26.98	109.72	80.87	27.82
GPR166	RKR	100.33	82.1	26.29	101.93	80.87	27.72
GPR173	RKR	113.16	83.16	24.89	115.12	90.91	29.04
GPR175	RKR	120.77	83.14	26.77	104.18	86.87	27.83
GPR176	RKR	124.85	81.15	24.64	113.24	88.91	28.48
GPR177	RKR	133.38	81.12	24.89	119.55	82.87	29.35
GPR194	RKR	134.02	98.93	26.73	136.82	91.87	30.61
GPR195	RKR	138.35	91.04	29.88	124.68	90.87	29.19
GPR196	RKR	132.78	92.19	27.91	123.81	92.91	27.81
GPR202	RKR	136.03	89.94	29.71	125.03	95.2	29.41

Genotype	Location	*Kharif* 2020			*Kharif* 2019		
		**PHT (cm)**	**DFF**	**PL (cm)**	**PHT (cm)**	**DFF**	**PL (cm)**
GPR206	RKR	119.19	102.16	28.52	94.96	107.87	27.38
GPR209	RKR	110.32	102.11	28.69	104.47	107.19	27.97
GPR213	RKR	115.49	88.44	28.23	106.19	106.87	28.23
GPR216	RKR	133.38	81.12	24.89	107.68	87.2	32.1
GPR222	RKR	118.86	81.19	30.81	108.18	91.2	30.48
GPR231	RKR	110.95	88.18	25.48	107.31	95.87	28.27
GPR234	RKR	99.15	90.18	26.64	90.2	107.19	24.82
GPR237	RKR	102.95	85.08	25.1	94.09	84.87	26.43
GPR238	RKR	95.1	84.1	25.81	92.25	93.87	25.52
GPR239	RKR	95.04	85.04	25.65	85.46	90.91	27.22
GPR240	RKR	95.36	81.3	26.69	100.07	87.87	30.07
GPR241	RKR	107.33	87.74	27.87	97.69	84.87	29.31
GPR247	RKR	111.4	88.03	28.12	107.12	90.87	29.46
GPR248	RKR	118	88.11	32.23	107.93	94.91	29.45
GPR251	RKR	107.02	90	30.36	103.64	82.87	29.77
GPR255	RKR	104.86	88.08	27.9	105.49	91.2	29.91
GPR258	RKR	113.16	88.09	27.51	106.27	88.87	29.06
GPR259	RKR	96.79	85.02	25.67	94.56	87.91	26.79
GPR260	RKR	113.35	91.79	27.17	96.86	88.91	29.54
GPR262	RKR	108.63	88.01	27.49	98.69	92.87	29.86
GPR263	RKR	103.62	89.92	27.49	97.14	94.91	27.45
GPR267	RKR	103.62	89.92	27.49	93.82	95.91	28.08
GPR270	RKR	113.85	86.18	28.05	94.73	85.87	27.2
GPR283	RKR	107.53	85.05	26.65	102.29	89.2	30.67
GPR290	RKR	108.45	86.12	27.5	101.05	91.2	27.23
GPR292	RKR	107.05	84.29	27.71	97.92	88.91	29.8
GPR293	RKR	120.23	84.26	28.38	100.16	90.91	28.55
GPR296	RKR	103.09	84.3	27.23	98.79	93.2	28.96
GPR298	RKR	111.58	84.25	27.08	104.37	92.87	28.78
GPR301	RKR	120.77	83.14	26.77	103.74	85.91	28.04
GPR303	RKR	107.78	82.13	26.98	104.51	93.87	28.68
GPR305	RKR	109.15	87.9	26.91	104.04	89.2	29.06
GPR308	RKR	104.51	87.89	27.48	106.43	85.2	28.26
GPR310	RKR	108.22	85.99	26.91	102.59	87.2	28.9
GPR312	RKR	127.74	81.23	27.58	103.07	82.91	28.19
GPR313	RKR	110.72	89.58	27.17	99.55	90.87	28.37
GPR314	RKR	109.99	90.88	27.29	109.26	90.87	27.76
GPR315	RKR	103.87	90.05	27.41	105.76	90.87	27.74
GPR319	RKR	113.85	86.18	28.05	106.07	89.2	27.12
GPR321	RKR	100.9	111.95	26.37	98.7	82.91	26.9
GPR324	RKR	100.75	88.36	26.4	106.1	98.91	26.95
GPR329	RKR	112.92	102.36	26.61	115.03	95.91	27.41
GPR331	RKR	105.92	112.88	25.82	95.26	96.87	26.88
GPR334	RKR	110.76	83.33	27.55	111.13	82.87	29.18

Genotype	Location	*Kharif* 2020			*Kharif* 2019		
		**PHT (cm)**	**DFF**	**PL (cm)**	**PHT (cm)**	**DFF**	**PL (cm)**
GPR335	RKR	97.47	92.96	28.16	100.26	87.87	27.25
GPR338	RKR	100.67	91.89	29.3	99.99	90.91	29.62
GPR339	RKR	109.15	87.9	26.91	111.8	90.91	29.78
GPR341	RKR	144.73	87.97	27.13	103.25	91.2	29.08
GPR360	RKR	102.26	90.81	27.39	97.97	86.87	27.81
GPM4	RKR	148.91	88.07	27.99	NA	81.03	28.23
GPM7	RKR	144.73	87.97	27.13	137.73	81.11	28.53
GPM11	RKR	158.25	84.04	26.56	135.96	81.03	27.57
GPM16	RKR	110.95	95.43	26.34	90.96	83.11	27.01
GPM20	RKR	103.88	94.54	26.69	91	85.03	27.98
GPM21	RKR	101.92	86.02	28.12	89.37	83.03	28.61
GPM22	RKR	101.92	86.02	28.12	95.5	83.03	28.58
GPM23	RKR	97.49	90.79	27.02	90.45	83.11	28.79
GPM25	RKR	127.43	112.86	26.68	105.12	100.11	28.27
GPM26	RKR	119.47	96.39	29.28	112.86	107.11	28.24
GPM27	RKR	126.53	110.41	30.22	104.33	102.03	28.01
GPM28	RKR	117.04	115.23	29.8	103.61	97.11	27.28
GPM29	RKR	114.32	114.29	29.77	106.42	103.11	26.83
GPM30	RKR	112.15	96.17	30.07	104.64	103.03	27.77
GPM33	RKR	119.29	96.27	29.57	101.85	113.11	27.8
GPM35	RKR	110	116	28.12	108.04	103.03	28.44
GPM36	RKR	116.39	90.59	28.47	102.62	93.11	28.59
GPM37	RKR	116.25	90.35	28.97	101.27	90.11	28.06
GPM40	RKR	122.99	95.33	28.2	102.53	87.03	29.44
GPM45	RKR	112.15	96.17	30.07	107.97	91.11	29.53
GPM48	RKR	133.25	111.83	29.86	106.46	114.03	27.78
GPM49	RKR	131.71	111.96	29.35	121.81	115.03	28.35
GPM53	RKR	124.49	113.8	28.89	110.61	115.03	29.04
GPM55	RKR	98.95	85.24	25.88	109.26	111.11	27.22
GPM60	RKR	100.54	84.08	25.84	110.32	109.03	26.62
GPM61	RKR	128.77	113.85	27.88	109.12	112.11	26.53
GPM71	RKR	134.23	114.77	28.39	115.64	112.11	27.26
GPM77	RKR	125.81	112.14	26.61	108.26	104.03	18.77
GPM81	RKR	128.41	105.15	29.64	119.56	115.03	28.27
GPM82	RKR	108.61	107.88	29.09	109.7	110.03	27.08
GPM83	RKR	115.41	100.16	28.54	85.09	108.03	26.72
GPM85	RKR	118.89	98.18	29.45	109.32	105.11	28.38
GPM86	RKR	118.99	96.23	28.22	97.29	107.11	27.29
GPM87	RKR	109.28	96.28	28.51	103.58	103.03	29.78
GPM88	RKR	96.43	98.25	27.28	118.2	103.11	28.38
GPM91	RKR	117.18	100.18	30.63	106.75	101.03	31.61
GPM93	RKR	118.97	100.21	27.05	101.1	105.03	28.7
GPM94	RKR	116.9	102.05	29.51	108.29	107.03	29.34
GPM98	RKR	113.64	113.04	27.54	109.84	89.11	30.93

Genotype	Location	*Kharif* 2020			*Kharif* 2019		
		**PHT (cm)**	**DFF**	**PL (cm)**	**PHT (cm)**	**DFF**	**PL (cm)**
GPM100	RKR	109.42	98.1	32.18	103.81	91.03	30.3
GPM101	RKR	109.45	101.28	31.52	105.23	107.03	31.38
GPM105	RKR	101.83	95.64	30.1	104.73	91.11	28.54
GPM106	RKR	104.49	96.34	31.3	104.45	98.03	32.15
GPM109	RKR	92.41	115.8	27.46	88.21	113.11	26.98
GPM113	RKR	83.7	115.68	27.61	113.87	NA	26.71
GPM114	RKR	91.84	113.66	27.44	81.21	113.11	25.57
GPM115	RKR	83.37	112.73	25.59	NA	111.03	24.64
GPM117	RKR	91.37	112.51	27.2	81.57	108.11	25.88
GPM118	RKR	94.75	111.81	26.52	86.29	112.03	25.62
GPM124	RKR	125.81	113.17	29.96	112.52	93.03	29.58
GPM127	RKR	124.83	88.34	28.59	116.59	105.11	28.79
P1401	RKR	94.05	111.15	27.1	93	107	29.75
ABL19	RKR	112.16	84.86	28.45	103.75	92	29.07
PB1509	RKR	103.39	83	27.06	91.49	83	26.71
P6B	RKR	97.59	82.68	24.7	86.95	92	23.84
PRR78	RKR	110.63	83.7	26.96	107.12	89.83	27.68
GPR3	MDP	117.72	86.99	28.2	98.82	94.16	27.7
GPR4	MDP	113.42	86.95	28.23	99.32	90.91	28.3
GPR7	MDP	147.75	82.9	31.66	108.81	86.16	29.24
GPR8	MDP	116.61	84.84	27.66	124.1	88.91	28.35
GPR19	MDP	121.7	91.1	29.54	95.49	90.16	28.2
GPR21	MDP	127.43	86.02	28	100.88	95.16	25.94
GPR23	MDP	120.46	82.86	29.94	101.68	88.91	27.66
GPR24	MDP	118.92	90.99	28.66	100.45	92.91	28.89
GPR32	MDP	108.32	85	26.08	90.1	90.16	26.91
GPR35	MDP	110.21	82.84	28.1	93.1	87.99	28.48
GPR37	MDP	102.1	82.87	27.85	95.54	85.99	29.65
GPR38	MDP	121.65	90.94	27.68	99.21	93.16	26.97
GPR39	MDP	130.29	90.98	29.41	96.67	92.91	26.82
GPR42	MDP	122.89	86.19	28.72	135.57	85.91	29.47
GPR43	MDP	107.33	80.98	NA	131.07	88.91	27.29
GPR45	MDP	124.29	83.01	26.16	103.32	90.16	27.19
GPR47	MDP	117.03	82.94	25.06	105.44	85.16	26.59
GPR52	MDP	114.24	84.14	23.54	101.62	87.91	26.51
GPR60	MDP	123.48	99.27	30.68	107.25	106.91	25.85
GPR62	MDP	123.48	99.26	29.99	100.47	104.99	27.66
GPR67	MDP	108.32	85	26.08	99.07	95.91	27.82
GPR70	MDP	122.94	84.93	28.22	100.51	93.91	27.6
GPR74	MDP	120.56	84.99	28.76	104.78	93.16	27.92
GPR77	MDP	113.37	86.07	30.48	101.57	93.91	27.36
GPR78	MDP	126.41	85.07	30.4	100.69	93.16	27.71
GPR80	MDP	115.71	84.97	29.91	108.15	92.91	27.83
GPR82	MDP	108.32	85	26.08	99.09	92.16	27.45

Genotype	Location	*Kharif* 2020			*Kharif* 2019		
		**PHT (cm)**	**DFF**	**PL (cm)**	**PHT (cm)**	**DFF**	**PL (cm)**
GPR86	MDP	115.71	84.97	29.91	104.31	92.99	28.83
GPR87	MDP	125.54	85.03	28.88	98.05	92.91	27.79
GPR92	MDP	121.57	85.06	28.41	104.3	93.16	28.05
GPR96	MDP	115.21	86.86	28.65	107.74	92.91	27.84
GPR100	MDP	122.07	86.87	28.19	103.57	92.16	29.75
GPR102	MDP	120.41	87.82	28.62	107.57	92.91	28.84
GPR104	MDP	123.71	86.88	28.25	102.67	89.99	27.29
GPR106	MDP	123.43	86.92	29.08	105.42	89.91	28.45
GPR111	MDP	115.09	94.87	27.53	86.11	85.99	25.88
GPR112	MDP	99.06	80.91	24.78	87.91	89.91	26.07
GPR113	MDP	114.87	92.97	27.95	94.6	96.91	26.72
GPR114	MDP	102.85	81.03	26.03	90.17	90.16	25.38
GPR115	MDP	107.5	82.21	26.7	101.74	NA	27.28
GPR117	MDP	112.43	93.12	28.05	88.56	96.91	26.34
GPR118	MDP	117.68	91.2	27.85	101.42	93.99	26.94
GPR119	MDP	119.56	91.05	28.81	94.62	96.16	26.46
GPR120	MDP	103.84	85.91	27.55	92.73	90.99	27.15
GPR131	MDP	120.37	100.17	31.23	100.83	103.15	28.57
GPR136	MDP	118.93	99.86	30.75	100.65	102.91	28.42
GPR142	MDP	125.83	85.98	28.93	96.51	94.91	26.68
GPR144	MDP	122.56	85.01	28.36	100.57	96.16	27.39
GPR146	MDP	123.83	85.97	28.65	104.12	92.99	26.87
GPR147	MDP	114.67	84.07	28.35	106.95	92.91	28.66
GPR148	MDP	126.08	85.11	29.12	104.34	94.16	27.91
GPR149	MDP	124.4	86.04	29.38	100.73	91.99	28.7
GPR150	MDP	121.57	85.06	28.41	100.9	91.91	28.34
GPR151	MDP	124.93	86.05	28.05	98.47	95.91	27.68
GPR155	MDP	110.85	83.13	25.87	98.06	89.91	26.77
GPR157	MDP	122.07	86.87	28.19	98.05	85.91	25.89
GPR160	MDP	116.44	87.02	25.36	92.55	86.91	26.9
GPR164	MDP	114.87	82.91	27.13	106.28	86.16	28.39
GPR166	MDP	117.05	82.89	26.68	103.74	83.16	28.68
GPR173	MDP	121.77	84.88	26.06	100.14	87.91	27.97
GPR175	MDP	128.79	82.04	28.1	104.2	86.16	28.29
GPR176	MDP	129.57	84.75	25.64	116.56	85.91	27.3
GPR177	MDP	134.73	84.73	25.49	114.55	85.91	28.85
GPR194	MDP	134.69	87.47	26.32	116.92	92.91	29.86
GPR195	MDP	127.71	89	30.86	118.62	89.91	29.37
GPR196	MDP	150.17	92.03	29.22	114.56	89.91	27.45
GPR202	MDP	126.41	85.07	30.4	121.53	97.99	28.71
GPR206	MDP	125.18	101.06	28.01	97.75	104.91	27.06
GPR209	MDP	121.63	101.01	29.53	96.71	104.99	27.75
GPR213	MDP	121.7	91.1	29.54	106.24	97.15	27.82
GPR216	MDP	120.01	83.79	NA	110.44	87.99	NA

Genotype	Location	*Kharif* 2020			*Kharif* 2019		
		**PHT (cm)**	**DFF**	**PL (cm)**	**PHT (cm)**	**DFF**	**PL (cm)**
GPR222	MDP	121.39	84.79	31.56	104.18	89.99	29.83
GPR231	MDP	117.03	89.9	25.78	98.73	99.15	27.21
GPR234	MDP	108.31	89.08	27.5	84.85	93.99	24.55
GPR237	MDP	112.41	85.86	25.7	92.14	88.16	25.91
GPR238	MDP	102.36	85.82	26.73	99.95	91.91	26.36
GPR239	MDP	102.36	85.82	26.73	93.29	90.91	27.49
GPR240	MDP	112.75	87.73	29.8	102.59	89.91	28.99
GPR241	MDP	102.1	82.87	27.85	97.47	87.91	28.7
GPR247	MDP	125.83	85.98	28.93	106.66	93.91	30.61
GPR248	MDP	123.74	88.89	32.37	110.8	94.91	29.59
GPR251	MDP	116.44	87.02	25.36	101.62	89.16	28.02
GPR255	MDP	116.17	86.98	28.64	102.61	92.99	29.33
GPR258	MDP	117.72	86.99	28.2	96.28	92.16	28.93
GPR259	MDP	109.45	84.86	26.12	93.44	88.91	26.5
GPR260	MDP	125.83	85.98	28.93	90.19	92.91	28.81
GPR262	MDP	112.24	87.85	28.85	98.84	94.91	29.84
GPR263	MDP	112.74	86.94	28.02	90.47	93.91	28.38
GPR267	MDP	112.74	86.94	28.02	95.69	92.91	28.34
GPR270	MDP	128.04	88.85	30.07	92.38	86.16	27.23
GPR283	MDP	124.25	85.83	27.81	100.59	89.99	29.56
GPR290	MDP	121.96	85.01	28.29	95.25	93.99	27.02
GPR292	MDP	112.51	87.89	29.04	94.87	93.91	28.76
GPR293	MDP	115.22	87.87	28.79	101.11	94.91	28.32
GPR296	MDP	116.95	87.91	28.41	97.89	90.99	28.69
GPR298	MDP	117	86.91	28.88	93.48	94.16	28.55
GPR301	MDP	109.45	84.86	26.12	102.39	91.91	27.51
GPR303	MDP	121.39	84.79	31.56	101.82	91.91	28.32
GPR305	MDP	106.37	84.92	28.92	96.72	89.99	28.51
GPR308	MDP	117.53	84.9	28.08	98.64	89.99	28.48
GPR310	MDP	117.86	84.89	28.16	107.15	89.99	29.67
GPR312	MDP	116.61	84.84	27.66	107.38	89.91	27.96
GPR313	MDP	127.2	78.12	27.95	100.58	90.16	28.83
GPR314	MDP	126.41	85.07	30.4	105.44	90.91	27.8
GPR315	MDP	108.96	85.18	28.19	102.86	93.91	27.45
GPR319	MDP	121.66	85.08	28.87	104.26	89.99	27.68
GPR321	MDP	110.71	85.44	27.07	103.03	89.91	26.67
GPR324	MDP	119.3	91.02	26.26	104.44	92.91	26.82
GPR329	MDP	113.61	86.2	26.7	96.73	91.91	26.98
GPR331	MDP	118.26	86.37	26.53	98.19	92.91	27.17
GPR334	MDP	112.74	86.94	28.02	106.07	93.16	29.03
GPR335	MDP	111.33	90.92	28.86	98.31	92.91	27.54
GPR338	MDP	116.86	86.08	30.26	97.15	89.91	30.71
GPR339	MDP	106.37	84.92	28.92	108.83	89.91	28.72
GPR341	MDP	153.7	84.99	27.67	104.83	87.99	28.54

Genotype	Location	*Kharif* 2020			*Kharif* 2019		
		PHT (cm)	DFF	PL (cm)	PHT (cm)	DFF	PL (cm)
GPR360	MDP	108.32	85	26.08	99.92	90.16	27.73
GPM4	MDP	NA	88.85	28.65	138.43	89.95	29.37
GPM7	MDP	NA	84.99	27.67	132.09	88.07	28.3
GPM11	MDP	NA	83.88	28.6	131.31	89.95	27.12
GPM16	MDP	110.46	85.86	28.53	90.29	91.07	26.98
GPM20	MDP	115.21	86.86	28.65	83.13	89.95	25.02
GPM21	MDP	106.37	84.92	28.92	91.8	92.95	27.79
GPM22	MDP	107.49	83.04	28.3	91.69	89.95	28.57
GPM23	MDP	101.77	83.11	27.42	88.36	93.07	27.86
GPM25	MDP	128.67	108.93	26.93	100.52	97.07	27.89
GPM26	MDP	140.31	100	30.62	109.4	102.07	27.52
GPM27	MDP	126.09	97.08	31.61	97.95	101.95	27.68
GPM28	MDP	117.11	94.36	29.25	96.75	95.07	26.93
GPM29	MDP	133.13	105.66	29.63	101.94	102.07	27.5
GPM30	MDP	119.47	96.01	31.6	97.75	99.95	27.32
GPM33	MDP	125.6	97.99	32.09	101.27	101.07	27.89
GPM35	MDP	110.41	NA	28.84	101.78	101.95	28.26
GPM36	MDP	121.69	98.9	29.34	101.98	93.07	28.23
GPM37	MDP	129.49	94.89	29.61	109.95	88.07	28.27
GPM40	MDP	128.63	NA	29.31	103.71	92.95	28.12
GPM45	MDP	126.24	96.95	29.69	117.19	86.07	29.86
GPM48	MDP	142.94	101.32	30.2	110.78	104.95	28.13
GPM49	MDP	139.54	106.15	30.31	113.97	108.95	28.15
GPM53	MDP	145.67	102.35	30.06	117.71	107.95	28.93
GPM55	MDP	145.11	105.78	29.5	121.46	109.07	27.46
GPM60	MDP	142.14	102.74	27.97	113.46	104.95	26.17
GPM61	MDP	144.37	106.16	29.48	116.88	110.07	27.54
GPM71	MDP	143.92	103.31	30.68	116.86	107.07	27.83
GPM77	MDP	138.88	104.45	29.21	112.58	106.95	24.23
GPM81	MDP	144.84	102.17	30.1	123.47	106.95	28.01
GPM82	MDP	152.73	100.19	29.78	113.08	104.95	27.12
GPM83	MDP	119.18	98.11	30.41	96.66	102.95	27.32
GPM85	MDP	126.09	97.08	31.61	106.03	100.07	28.16
GPM86	MDP	120.4	96.07	29.54	103.43	102.07	28.17
GPM87	MDP	125.04	96.12	30.61	107.3	101.95	29.58
GPM88	MDP	106.62	98.09	28.13	98.03	102.07	27.79
GPM91	MDP	122.98	100.96	31.01	106.6	102.95	31.07
GPM93	MDP	128.14	99.11	27.98	97.74	102.95	27.76
GPM94	MDP	126.58	99.07	30.12	107.87	104.95	30.71
GPM98	MDP	116.06	NA	29.25	101.52	94.07	30.54
GPM100	MDP	130.92	102.64	34.91	104.61	101.95	30.9
GPM101	MDP	118.46	105.82	32.76	104.05	95.95	31.43
GPM105	MDP	113.37	86.07	30.48	96.77	97.07	28.69
GPM106	MDP	124.14	99.94	32.3	98.24	96.95	31.52

Genotype	Location	*Kharif* 2020			*Kharif* 2019		
		**PHT (cm)**	**DFF**	**PL (cm)**	**PHT (cm)**	**DFF**	**PL (cm)**
GPM109	MDP	108.67	107.17	29.33	86.22	108.07	26.48
GPM113	MDP	94.05	107.99	28.23	80.51	112.07	25.5
GPM114	MDP	104.56	106.91	28.21	86.05	109.07	26.44
GPM115	MDP	95.37	105.05	26.68	78.74	96.95	24.9
GPM117	MDP	100.04	104.83	27.5	86.45	108.07	26.64
GPM118	MDP	106.8	103.18	27.37	81.67	106.95	25.91
GPM124	MDP	124.85	91.36	31.2	108.17	98.95	28.64
GPM127	MDP	128.94	91	30.85	106.21	99.07	28.32
P1401	MDP	110.32	104.86	28.54	90.45	105.16	28.77
ABL19	MDP	117.68	85.98	29.08	96.89	95	27.88
PB1509	MDP	111.36	81.82	27.31	99.51	85.33	28.2
P6B	MDP	106.81	85.13	25.02	85.83	96.83	23.75
PRR78	MDP	114.02	85.49	27.32	102.66	94.5	27.67
GPR3	KNL	112.87	88.94	26.95	98.85	92.93	26.89
GPR4	KNL	110.7	87.96	26.51	98.35	93.05	28.59
GPR7	KNL	158.04	76.38	29.39	117.08	89.93	29.3
GPR8	KNL	120.38	81.14	27.24	116.14	86.05	28.08
GPR19	KNL	114.98	97.76	28.05	96.27	93.93	27.71
GPR21	KNL	121.67	86.09	26.67	100.12	89.93	26.21
GPR23	KNL	114.37	78.22	28.22	95.86	92.21	28.14
GPR24	KNL	113.08	95.76	29.05	98.9	94.21	28.38
GPR32	KNL	98.67	89.77	24.76	90.91	91.93	26.82
GPR35	KNL	110.53	76.32	27.4	92.86	90.87	28.34
GPR37	KNL	104.78	81.06	28.2	93.81	84.87	29.5
GPR38	KNL	96.3	98.53	24.9	98.56	93.93	27.83
GPR39	KNL	115.45	95.75	28.39	98.64	97.05	26.32
GPR42	KNL	108.02	97.55	26.53	NA	88.21	30.29
GPR43	KNL	114.33	77.28	22.65	104.89	89.21	26.14
GPR45	KNL	116.51	85.9	25.15	99.29	93.93	27.43
GPR47	KNL	119.72	83.01	23.58	94.18	85.93	25.36
GPR52	KNL	112.62	82.33	22.06	97.21	88.21	26.03
GPR60	KNL	115.35	107.81	29.19	104.07	108.05	26.88
GPR62	KNL	113.44	106.86	28.82	99.35	111.87	27.29
GPR67	KNL	114.9	86.01	26.27	98.29	91.21	28.38
GPR70	KNL	111.61	83.12	26.19	95.6	89.05	28.48
GPR74	KNL	111.42	85.05	27.28	99.73	93.93	28.32
GPR77	KNL	119.57	86.13	29.43	95.38	93.05	27.33
GPR78	KNL	111.71	86.08	26.18	100.28	88.93	27.99
GPR80	KNL	112.79	80.34	25.1	95.94	93.05	27.88
GPR82	KNL	108.58	82.25	26.66	97.02	92.93	26.75
GPR86	KNL	112.79	80.34	25.1	98.35	NA	28.63
GPR87	KNL	107.28	86.98	26.07	94.74	92.05	27.41
GPR92	KNL	109.85	87.95	26.01	100.95	93.93	27.66
GPR96	KNL	111.88	85.98	30	103.41	91.21	28.44

Genotype	Location	*Kharif* 2020			*Kharif* 2019		
		PHT (cm)	DFF	PL (cm)	PHT (cm)	DFF	PL (cm)
GPR100	KNL	111.86	86.94	26.37	100.04	95.93	29.66
GPR102	KNL	108.09	86.95	27.26	99.72	103.21	28.51
GPR104	KNL	114.9	86.01	26.27	99.37	88.87	27.03
GPR106	KNL	109.25	86.05	26.63	101.7	92.05	28.14
GPR111	KNL	104.8	91.17	24.45	85.59	90.87	25.25
GPR112	KNL	101.76	80.03	23.12	86.64	86.05	26.26
GPR113	KNL	108.46	94.92	26.28	89.05	101.21	26.19
GPR114	KNL	96.32	81.1	25.61	86.03	88.93	24.57
GPR115	KNL	116.97	91.68	25.82	95.24	101.21	26.51
GPR117	KNL	110.75	97.89	25.99	91.41	103.21	27.17
GPR118	KNL	113.13	98.79	26.76	95.38	98.87	26.13
GPR119	KNL	116.19	94.88	26.86	95.26	100.93	26.18
GPR120	KNL	112.79	80.34	25.1	89.23	88.87	27.63
GPR131	KNL	111.59	102.12	29.68	97.81	110.92	28.25
GPR136	KNL	106.11	102.75	29.32	96.8	111.05	28.93
GPR142	KNL	114.18	84.17	27.42	99.4	94.21	26.97
GPR144	KNL	114.8	86.02	26.53	99.18	97.93	27.06
GPR146	KNL	115.22	84.15	27.18	95.88	90.87	27.19
GPR147	KNL	119.57	80.38	26.83	100.82	89.21	27.78
GPR148	KNL	116.97	83.3	28.16	98.98	93.93	27.63
GPR149	KNL	110.39	87.05	27.59	101.43	86.87	29.03
GPR150	KNL	113.47	87.01	26.5	94	92.05	27.53
GPR151	KNL	117.85	86.12	26.92	99.43	91.21	27.64
GPR155	KNL	108.49	91.67	25.14	92.79	88.21	26.64
GPR157	KNL	117.88	84.11	25.1	96.43	92.21	26.3
GPR160	KNL	106.32	91.79	24.24	95.7	86.05	27.08
GPR164	KNL	121.72	79.22	24.88	101.23	84.93	28.4
GPR166	KNL	111.65	77.31	24.07	100.45	87.93	28.14
GPR173	KNL	115.36	79.3	25.18	101.84	90.05	28.23
GPR175	KNL	131.33	81.17	26.74	100.03	86.93	27.93
GPR176	KNL	112.28	78.23	24.7	110.31	90.05	26.92
GPR177	KNL	119.92	78.21	23.81	106.91	88.21	28.64
GPR194	KNL	128.44	109.19	26.71	118.21	90.21	30.94
GPR195	KNL	132.83	90.01	28.94	119.35	89.21	29.27
GPR196	KNL	140.6	92.1	28.72	113.87	97.05	27.66
GPR202	KNL	134.82	88.9	28.6	115.52	94.87	28.45
GPR206	KNL	116.79	105.83	28.21	102.1	110.21	26.53
GPR209	KNL	104.29	103.9	28.95	97.59	104.87	28.18
GPR213	KNL	121.62	98.7	28.25	103.93	104.93	27.44
GPR216	KNL	103.34	75.38	25.16	103.92	81.88	NA
GPR222	KNL	110.8	76.39	28.78	104.28	88.87	30.58
GPR231	KNL	108.25	88.08	24.49	98.82	100.93	27.45
GPR234	KNL	100.93	93.85	26.21	88.33	104.87	25.34
GPR237	KNL	121.07	79.34	23.24	89.8	87.93	26.01

Genotype	Location	*Kharif* 2020			*Kharif* 2019		
		PHT (cm)	DFF	PL (cm)	PHT (cm)	DFF	PL (cm)
GPR238	KNL	93.29	80.24	24.74	92.54	89.21	25.6
GPR239	KNL	115.36	79.3	25.18	86.93	91.05	26.86
GPR240	KNL	119.47	82.15	28.98	97.32	89.21	29.04
GPR241	KNL	98.77	75.41	26.33	94.53	89.21	28.51
GPR247	KNL	109.25	86.05	26.63	100.84	93.21	29.6
GPR248	KNL	119.57	86.13	29.43	101.83	95.05	30.3
GPR251	KNL	122.1	89.91	30.19	98.13	88.93	29.37
GPR255	KNL	115.21	88.93	26.88	104.8	91.87	28.83
GPR258	KNL	112.87	88.94	26.95	99.63	91.93	29.37
GPR259	KNL	100.38	79.28	25.03	90.4	88.05	26.43
GPR260	KNL	119.19	81.35	25.86	97.45	91.05	29.66
GPR262	KNL	115.24	80.39	26.7	99.19	97.21	29.2
GPR263	KNL	107.39	81.36	28.02	95.56	97.05	28.2
GPR267	KNL	110.5	83.24	27.52	90.17	91.05	28.15
GPR270	KNL	121.67	86.09	26.67	95.11	87.93	28.08
GPR283	KNL	115.1	77.43	26.68	95.47	88.87	29.75
GPR290	KNL	114.8	86.02	26.53	96.19	89.87	27.26
GPR292	KNL	110.7	87.96	26.51	92.78	96.05	28.3
GPR293	KNL	108.39	86.99	26.53	93.97	97.05	27.44
GPR296	KNL	109.75	88.92	25.91	99	90.87	29.48
GPR298	KNL	109.92	86.04	26.43	96.83	94.93	28.52
GPR301	KNL	100.38	79.28	25.03	98.91	96.05	27.66
GPR303	KNL	116.61	80.16	25.45	98.17	91.21	27.8
GPR305	KNL	117.5	79.34	26.37	100.41	90.87	28.75
GPR308	KNL	110.83	79.33	26.65	102.19	90.87	27.61
GPR310	KNL	110.31	80.25	25.71	99.5	82.87	29.34
GPR312	KNL	120.38	81.14	27.24	99.05	86.05	28.07
GPR313	KNL	130.63	75.37	26.44	97.79	89.93	28.8
GPR314	KNL	112.25	85.14	26.17	102.88	91.21	27.16
GPR315	KNL	102.54	94.66	25.39	101.92	93.21	27.34
GPR319	KNL	106.17	90.8	27.16	109.07	90.87	27.61
GPR321	KNL	114.06	93.04	26.03	96.85	92.05	26.91
GPR324	KNL	110.4	96.73	25.38	95.71	97.05	26.51
GPR329	KNL	129.87	86.27	25.42	98.77	102.05	27.44
GPR331	KNL	121.35	90.2	25.79	94.67	99.21	27.66
GPR334	KNL	122.65	88.89	26.71	104.67	87.93	28.76
GPR335	KNL	109.36	88.17	28.17	91.14	89.21	27.95
GPR338	KNL	111.34	88.03	28.12	94.48	97.05	29.66
GPR339	KNL	121.73	77.46	26.67	102.4	91.05	29.17
GPR341	KNL	113.77	81.29	26.19	98.35	NA	28.72
GPR360	KNL	114.9	86.01	26.27	97.61	90.93	27.91
GPM4	KNL	130.61	85.15	28.32	NA	90.97	27.62
GPM7	KNL	135.12	82.23	27.69	129.42	88.01	27.64
GPM11	KNL	138.65	82.07	27.79	130.42	90.97	27.53

Genotype	Location	*Kharif* 2020			*Kharif* 2019		
		PHT (cm)	DFF	PL (cm)	PHT (cm)	DFF	PL (cm)
GPM16	KNL	98.63	78.4	26.23	91.59	91.01	27.12
GPM20	KNL	116.78	77.51	27.53	86.16	90.97	27.42
GPM21	KNL	110.56	86.87	27.89	92.81	82.97	28.38
GPM22	KNL	110.56	86.87	27.89	91.21	80.97	28.22
GPM23	KNL	103.37	86	26.08	88.85	87.01	28.25
GPM25	KNL	113.61	110.88	25.27	106.7	99.01	26.84
GPM26	KNL	121.7	108.53	27.72	106.41	103.01	27.85
GPM27	KNL	120.37	99.03	29.8	98.68	104.97	27.89
GPM28	KNL	112.35	108.55	28.76	100.31	97.01	26.83
GPM29	KNL	123.98	96.31	28.83	101.43	105.01	26.96
GPM30	KNL	105.25	100.78	29	99.4	102.97	26.33
GPM33	KNL	107.84	97.12	27.26	104.25	113.01	28.41
GPM35	KNL	107.67	114.96	28.2	98.27	108.97	27.89
GPM36	KNL	107.18	89.56	28.04	98.81	94.01	28.4
GPM37	KNL	122.78	96.84	29.12	101.36	90.01	28.78
GPM40	KNL	131.47	99.94	28.32	96.45	100.97	27.88
GPM45	KNL	130.98	99.84	27.25	103.73	91.01	29.32
GPM48	KNL	133.29	112.68	29.21	103.56	113.96	27.66
GPM49	KNL	131.06	112.81	28.73	109.52	112.96	28.11
GPM53	KNL	130.43	113.71	28.09	106.12	113.96	28.22
GPM55	KNL	127.86	113.38	27.45	107.45	113.01	27.54
GPM60	KNL	127.08	107.51	26.78	105.9	111.96	26.68
GPM61	KNL	128.97	112.82	27.9	107.62	113.01	27.06
GPM71	KNL	126.16	112.79	27.92	108.28	110.01	27.11
GPM77	KNL	126.52	NA	27.55	105.18	113.96	23.84
GPM81	KNL	129.11	111.65	27.79	108.22	114.96	27.72
GPM82	KNL	129.74	106.84	28.49	109.99	106.97	27.08
GPM83	KNL	109.18	106.65	29.11	94.72	107.97	27.26
GPM85	KNL	118.07	104.67	28.46	103.15	105.01	28.62
GPM86	KNL	120.03	103.66	28.75	100.94	105.01	27.76
GPM87	KNL	109.47	105.6	28.85	107.15	96.97	29.6
GPM88	KNL	104.9	106.62	27.86	101.13	105.01	27.87
GPM91	KNL	113.33	108.56	29.56	104.49	104.97	31.34
GPM93	KNL	124.4	109.53	27	99.83	104.97	27.54
GPM94	KNL	121.32	105.72	29.18	102.1	106.97	29.61
GPM98	KNL	115.86	110.12	28.43	101.03	90.01	31.66
GPM100	KNL	113.56	103.65	30.45	98.98	90.97	30.31
GPM101	KNL	115.73	107.77	30.64	98.55	88.97	31.33
GPM105	KNL	117.06	81.43	29.93	98.58	93.01	29.88
GPM106	KNL	117.35	105.66	30.46	99.45	101.97	31.91
GPM109	KNL	97.16	NA	26.86	88.06	115.01	26.66
GPM113	KNL	86.14	112.76	26.74	90.11	113.01	25.46
GPM114	KNL	89.16	107.92	26.06	88.04	113.01	25.66
GPM115	KNL	84.99	110.76	25.58	NA	112.96	24.98

**Genotype**	**Location**	***Kharif* 2020**			***Kharif* 2019**		
		**PHT (cm)**	**DFF**	**PL (cm)**	**PHT (cm)**	**DFF**	**PL (cm)**
GPM117	KNL	102.37	99.25	27.62	88.84	111.01	26.85
GPM118	KNL	98.66	108.89	25.53	NA	111.96	25.87
GPM124	KNL	120.6	96.13	29.67	110.67	98.97	29.16
GPM127	KNL	123.84	98.6	28.15	107.34	103.01	28.15
P1401	KNL	97.73	114.44	27.16	92.9	110.84	29.83
ABL19	KNL	113.16	82.37	27.8	97.66	93.67	28.64
PB1509	KNL	110.6	77.71	24.71	89.58	85.67	26.9
P6B	KNL	106.81	78.05	24.37	87.14	88.84	24.91
PRR78	KNL	112.07	85.33	26.42	100.3	92.01	27.58
GPR3	DEL	108.04	90.04	29.47			
GPR4	DEL	105.66	88.11	28.49			
GPR7	DEL	149.62	84.06	32.02			
GPR8	DEL	135.11	86	28.74			
GPR19	DEL	110.64	95.09	29.35			
GPR21	DEL	118.32	89.07	28.2			
GPR23	DEL	123.05	84.96	31.32			
GPR24	DEL	108.46	94.04	29.33			
GPR32	DEL	94.81	85.22	26.4			
GPR35	DEL	104.98	84.01	28.46			
GPR37	DEL	95.64	82.15	27.97			
GPR38	DEL	115.49	87.39	27.96			
GPR39	DEL	116.78	92.14	30.47			
GPR42	DEL	116.15	88.29	28.85			
GPR43	DEL	99.24	85.9	23.42			
GPR45	DEL	107.97	86.99	28.31			
GPR47	DEL	158.43	83.16	28.66			
GPR52	DEL	109.86	93.77	24.69			
GPR60	DEL	106.55	109.85	32.25			
GPR62	DEL	114.53	109.83	30.2			
GPR67	DEL	115.14	87.1	28.9			
GPR70	DEL	111.46	87.98	28.66			
GPR74	DEL	113.79	90.86	28.47			
GPR77	DEL	102.56	88.17	29.33			
GPR78	DEL	105.66	88.11	28.49			
GPR80	DEL	96.1	88.02	27.98			
GPR82	DEL	106.76	88.99	30.4			
GPR86	DEL	108.97	88.96	29.29			
GPR87	DEL	104.9	88.07	28.52			
GPR92	DEL	109.61	89.04	29.07			
GPR96	DEL	105.17	89.9	28.77			
GPR100	DEL	157.99	89.91	28.13			
GPR102	DEL	106.76	88.99	30.4			
GPR104	DEL	106.76	88.99	30.4			
GPR106	DEL	105.9	91.85	28.57			

Genotype	Location	*Kharif* 2020			*Kharif* 2019		
		**PHT (cm)**	**DFF**	**PL (cm)**	**PHT (cm)**	**DFF**	**PL (cm)**
GPR111	DEL	103.97	100.73	27.31			
GPR112	DEL	93.68	79.25	24.53			
GPR113	DEL	107.47	99.78	27.67			
GPR114	DEL	91.19	83.13	27.03			
GPR115	DEL	114.16	83.37	26.97			
GPR117	DEL	109.88	99.93	28.14			
GPR118	DEL	121.13	99.89	29.21			
GPR119	DEL	113.51	96.91	29.16			
GPR120	DEL	96.1	88.02	27.98			
GPR131	DEL	106.72	108.87	31.19			
GPR136	DEL	109	108.55	30.87			
GPR142	DEL	112.01	89.03	29.2			
GPR144	DEL	112.46	88.06	28.47			
GPR146	DEL	107.98	89.96	27.99			
GPR147	DEL	108.28	90.88	30.03			
GPR148	DEL	108.04	90.04	29.47			
GPR149	DEL	108.72	90.02	29.57			
GPR150	DEL	109.61	89.04	29.07			
GPR151	DEL	108.04	90.04	29.47			
GPR155	DEL	105.13	87.12	26.84			
GPR157	DEL	113.18	88.97	26.79			
GPR160	DEL	106.68	90.07	25.61			
GPR164	DEL	106.46	86.9	27.64			
GPR166	DEL	102.38	86.87	27.48			
GPR173	DEL	123.32	89.8	26.8			
GPR175	DEL	119.02	89.79	28.53			
GPR176	DEL	130.04	84.03	24.38			
GPR177	DEL	134.69	82.12	26.34			
GPR194	DEL	148.16	93.34	28.26			
GPR195	DEL	149.45	92.98	31.08			
GPR196	DEL	148.45	101.66	29.99			
GPR202	DEL	133.63	87.17	30.46			
GPR206	DEL	121.43	109.75	29.17			
GPR209	DEL	122.61	107.82	30.3			
GPR213	DEL	103.97	100.73	27.31			
GPR216	DEL	117.94	85.89	28.51			
GPR222	DEL	110.55	88.78	30.68			
GPR231	DEL	116.83	93.88	26.23			
GPR234	DEL	103.88	95.89	27.17			
GPR237	DEL	100.72	89.85	25.77			
GPR238	DEL	96.92	86.98	26.49			
GPR239	DEL	96.92	86.98	26.49			
GPR240	DEL	104.95	87.95	29.47			
GPR241	DEL	95.64	82.15	27.97			

Genotype	Location	*Kharif* 2020			*Kharif* 2019		
		**PHT (cm)**	**DFF**	**PL (cm)**	**PHT (cm)**	**DFF**	**PL (cm)**
GPR247	DEL	112.01	89.03	29.2			
GPR248	DEL	110.29	91.93	32.53			
GPR251	DEL	113.06	89.12	31.78			
GPR255	DEL	102.29	89.08	28.86			
GPR258	DEL	108.04	90.04	29.47			
GPR259	DEL	99.79	86.97	25.96			
GPR260	DEL	119.1	89.97	29.56			
GPR262	DEL	95.99	91.84	28.76			
GPR263	DEL	99.02	91.86	29.34			
GPR267	DEL	101.97	89.98	29.97			
GPR270	DEL	115	90.95	30.06			
GPR283	DEL	104.96	89.82	28.58			
GPR290	DEL	104.19	89.94	28.82			
GPR292	DEL	106.16	90.94	29.26			
GPR293	DEL	119.1	89.97	29.56			
GPR296	DEL	115	90.95	30.06			
GPR298	DEL	105.6	90.9	29.03			
GPR301	DEL	108.54	88.85	28.12			
GPR303	DEL	106.08	84.08	28.05			
GPR305	DEL	103.84	87.96	28.03			
GPR308	DEL	102.41	87.01	29.09			
GPR310	DEL	107.97	86.99	28.31			
GPR312	DEL	110.77	86.94	28.59			
GPR313	DEL	105.66	82.11	28.25			
GPR314	DEL	109.78	90	29.56			
GPR315	DEL	97.38	89.17	28.31			
GPR319	DEL	NA	90.01	29.55			
GPR321	DEL	98.29	87.54	27.45			
GPR324	DEL	107.05	96.89	27.04			
GPR329	DEL	129.55	90.19	27.79			
GPR331	DEL	105.33	87.53	27.12			
GPR334	DEL	111.69	90.92	28.44			
GPR335	DEL	104.31	93.97	28.85			
GPR338	DEL	106.68	90.07	25.61			
GPR339	DEL	100.72	89.85	25.77			
GPR341	DEL	113.79	90.86	28.47			
GPR360	DEL	104.55	84.28	28.38			
GPM4	DEL	NA	90.01	29.55			
GPM7	DEL	157.99	89.91	28.13			
GPM11	DEL	158.43	83.16	28.66			
GPM16	DEL	101.58	80.43	27.27			
GPM20	DEL	105.32	85.2	28.01			
GPM21	DEL	96.26	82.32	28.85			
GPM22	DEL	95.34	84.2	27.69			

Genotype	Location	*Kharif* 2020			*Kharif* 2019		
		**PHT (cm)**	**DFF**	**PL (cm)**	**PHT (cm)**	**DFF**	**PL (cm)**
GPM23	DEL	97.23	85.21	27.67			
GPM25	DEL	121.09	111.04	28.04			
GPM26	DEL	122.93	104.92	29.94			
GPM27	DEL	116.31	96.36	31.78			
GPM28	DEL	109.02	92.7	30.51			
GPM29	DEL	130.61	94.59	30.58			
GPM30	DEL	113.91	100.94	30.98			
GPM33	DEL	123.09	109.51	30.7			
GPM35	DEL	105.36	123.59	28.62			
GPM36	DEL	115.63	119.83	28.73			
GPM37	DEL	121.4	98.88	29.86			
GPM40	DEL	125.26	95.39	30.73			
GPM45	DEL	113.91	100.94	30.98			
GPM48	DEL	135.53	111.9	30.85			
GPM49	DEL	141.92	115.78	30.24			
GPM53	DEL	130.82	113.86	30.39			
GPM55	DEL	129.93	115.41	28.96			
GPM60	DEL	134.05	109.55	28.69			
GPM61	DEL	140.33	114.86	29.18			
GPM71	DEL	134.99	114.83	30.38			
GPM77	DEL	134.67	122.55	29.14			
GPM81	DEL	136.19	111.8	28.94			
GPM82	DEL	132.09	108.88	29.65			
GPM83	DEL	122.93	104.92	29.94			
GPM85	DEL	119.91	102.94	30.58			
GPM86	DEL	114.27	101.94	30.42			
GPM87	DEL	108.61	103.87	30.48			
GPM88	DEL	96.95	104.9	28.7			
GPM91	DEL	116.9	102.13	32.28			
GPM93	DEL	129.79	104.98	28.31			
GPM94	DEL	121.31	104	28.31			
GPM98	DEL	106.05	120.63	28.02			
GPM100	DEL	111.97	92.51	34.15			
GPM101	DEL	108.96	107.93	33.17			
GPM105	DEL	110.29	91.93	32.53			
GPM106	DEL	114.3	103.93	33.57			
GPM109	DEL	102.81	109.28	29.04			
GPM113	DEL	96.71	107.27	27.86			
GPM114	DEL	99.62	105.25	28.63			
GPM115	DEL	96.05	106.21	27.72			
GPM117	DEL	97.17	102.22	29.64			
GPM118	DEL	95.61	111.87	27.15			
GPM124	DEL	127.85	97.23	31.22			
GPM127	DEL	127.04	94.05	30.87			

Genotype	Location	*Kharif* 2020			*Kharif* 2019		
		PHT (cm)	DFF	PL (cm)	PHT (cm)	DFF	PL (cm)
P1401	DEL	100.64	110.51	27.66			
ABL19	DEL	104.81	89.82	29.72			
PB1509	DEL	100.69	85.16	27.75			
P6B	DEL	100.59	84.18	25.89			
PRR78	DEL	106.97	88.83	28.5			

. DFF, Days to fifty percent flowering; PHT, Plant height in cm; PL, Panicle length in cm;

Supplementary Table 5: BLUE values of KLBC, KLAC, and LBR of 172 breeding lines over 4 environments

Genotype	Environment	KLBC	KLAC	LBR	Environment	KLBC	KLAC	LBR
GPR3	DEL20	7.99	13.19	4.98	MDP20	8.04	15.13	5.08
GPR4	DEL20	8.17	13.26	4.97	MDP20	8.06	13.17	4.79
GPR7	DEL20	6.29	9.01	3.86	MDP20	6.43	9.13	3.56
GPR8	DEL20	8.64	13.57	5.7	MDP20	7.97	14.43	4.72
GPR19	DEL20	8.12	16.1	4.68	MDP20	8.24	16.08	5.27
GPR21	DEL20	6.72	11.48	3.83	MDP20	6.6	13.66	3.77
GPR23	DEL20	7.97	13.87	5.32	MDP20	8.33	14.82	4.74
GPR24	DEL20	7.98	15.3	4.43	MDP20	8.9	17.2	5.22
GPR32	DEL20	8.13	16.42	4.64	MDP20	8	14.12	4.64
GPR35	DEL20	8.02	15.13	4.69	MDP20	8.38	14.96	5.13
GPR37	DEL20	8.4	14.13	4.87	MDP20	8.18	15.34	4.73
GPR38	DEL20	8.19	14.51	4.84	MDP20	8.46	16.37	4.81
GPR39	DEL20	8.43	15.68	4.62	MDP20	8.06	16.39	4.86
GPR42	DEL20	8.29	13.94	5.07	MDP20	8.22	11.92	4.32
GPR43	DEL20	5.66	10.45	2.97	MDP20	5.82	11.43	3.26
GPR45	DEL20	7.22	14.71	3.68	MDP20	7.79	14.69	4.11
GPR47	DEL20	8.32	16.72	5.38	MDP20	8.88	15.32	5.08
GPR52	DEL20	8.4	15.13	4.97	MDP20	8.71	16.83	5.15
GPR60	DEL20	8.13	15.89	4.71	MDP20	7.95	15.87	4.73
GPR62	DEL20	8.25	14.47	5.22	MDP20	7.76	16.1	4.98
GPR67	DEL20	7.97	14.24	4.73	MDP20	7.96	14.11	4.99
GPR70	DEL20	8.01	13.63	4.76	MDP20	8.06	14.79	5.06
GPR74	DEL20	8.38	14.81	4.96	MDP20	8.07	14.1	4.8
GPR77	DEL20	7.99	13.78	4.79	MDP20	8.23	13.87	4.7
GPR78	DEL20	8.17	14.94	4.8	MDP20	7.88	14.59	4.63
GPR80	DEL20	8.62	14.9	5.04	MDP20	7.98	14.44	4.75
GPR82	DEL20	8.35	14.31	4.84	MDP20	8.1	15.47	4.75
GPR86	DEL20	8.58	13.92	4.95	MDP20	8.17	15.22	4.71
GPR87	DEL20	8.43	14.31	5.12	MDP20	8.68	14.89	4.9

Genotype	Environment	KLBC	KLAC	LBR	Environment	KLBC	KLAC	LBR
GPR92	DEL20	8.11	14.01	4.82	MDP20	8.23	14.62	4.88
GPR96	DEL20	8.66	14.25	5.09	MDP20	8.44	14.52	5.27
GPR100	DEL20	8.38	14.1	4.82	MDP20	8.37	13.92	4.89
GPR102	DEL20	8.42	14.51	4.95	MDP20	8.14	15.05	4.78
GPR104	DEL20	7.96	14.54	4.75	MDP20	8.25	14.88	4.81
GPR106	DEL20	7.84	14.57	4.88	MDP20	8.34	15.99	4.91
GPR111	DEL20	8.44	17.01	5.21	MDP20	8.53	17.16	5.48
GPR112	DEL20	8.23	16.27	4.86	MDP20	8.1	16.15	4.81
GPR113	DEL20	7.89	16.51	4.96	MDP20	8.3	16.2	5.19
GPR114	DEL20	7.75	15.77	4.83	MDP20	8.05	16.66	5.11
GPR115	DEL20	8.22	15.75	5.03	MDP20	8.28	16.09	5.02
GPR117	DEL20	8.5	16.73	5.19	MDP20	8.17	16.92	5.07
GPR118	DEL20	8.59	15.83	5.1	MDP20	8.3	17.05	5.07
GPR119	DEL20	8.29	16.87	4.88	MDP20	8.3	16.43	5.02
GPR120	DEL20	7.76	13.56	4.96	MDP20	7.58	13.56	4.64
GPR131	DEL20	7.56	14.16	5.02	MDP20	7.57	15.45	5.19
GPR136	DEL20	7.53	15.1	4.95	MDP20	7.54	15.45	5.01
GPR142	DEL20	7.88	15.4	4.72	MDP20	8.2	15.19	4.95
GPR144	DEL20	7.94	13.31	4.87	MDP20	8.03	14.24	4.91
GPR146	DEL20	8.02	14.73	4.8	MDP20	8.42	15.28	5.09
GPR147	DEL20	8.08	14.02	5.03	MDP20	8.31	14.9	4.79
GPR148	DEL20	7.86	14.33	4.8	MDP20	8.46	13.52	5.08
GPR149	DEL20	8.2	14.08	5.33	MDP20	8.49	14.64	4.96
GPR150	DEL20	8.14	14.17	4.84	MDP20	8.24	14.73	4.88
GPR151	DEL20	7.89	14.58	4.56	MDP20	8.03	15.89	4.83
GPR155	DEL20	7.6	14.31	4.46	MDP20	8.54	16.27	5.05
GPR157	DEL20	7.95	14.33	4.6	MDP20	8.74	16.18	5.09
GPR160	DEL20	8.3	14.06	4.88	MDP20	7.84	15.37	4.85
GPR164	DEL20	7.76	14.68	5.38	MDP20	8.64	15.18	NA
GPR166	DEL20	7.19	12.68	4.34	MDP20	7.13	15.67	4.56
GPR173	DEL20	7.51	14.99	4.43	MDP20	7.71	15.58	4.72
GPR175	DEL20	7.76	16.58	4.71	MDP20	8.34	16.18	5.09
GPR176	DEL20	8.54	15.31	5.16	MDP20	8.52	17.7	5.4
GPR177	DEL20	8.29	15.13	5.05	MDP20	8.24	16.02	5
GPR194	DEL20	7.09	12.16	4.46	MDP20	7.17	12.09	4.11
GPR195	DEL20	9.01	16.63	5.23	MDP20	9.15	15.28	5.22
GPR196	DEL20	8.99	14.99	5.25	MDP20	9	15.34	5.51
GPR202	DEL20	8.11	16.48	4.76	MDP20	8.35	16.78	4.71
GPR206	DEL20	7.7	14.06	4.58	MDP20	8.35	14.87	4.67

Genotype	Environment	KLBC	KLAC	LBR	Environment	KLBC	KLAC	LBR
GPR209	DEL20	7.77	15.43	4.47	MDP20	8.54	16.19	4.64
GPR213	DEL20	8.9	15.9	4.77	MDP20	8.67	15.29	4.76
GPR216	DEL20	8.75	15.51	5.46	MDP20	9.26	14.53	5.53
GPR222	DEL20	9.04	15.48	5.57	MDP20	9.21	14.04	5.56
GPR231	DEL20	8.61	14.47	5.45	MDP20	8.04	15.92	4.61
GPR234	DEL20	8.52	13.18	5.14	MDP20	8.98	15.87	5.35
GPR237	DEL20	8.75	15.78	5.48	MDP20	8.78	15.67	5.06
GPR238	DEL20	8.19	15.97	5.47	MDP20	8.17	14.9	4.66
GPR239	DEL20	8.6	16.33	5.38	MDP20	8.9	16.35	5.02
GPR240	DEL20	8.68	16.6	5.25	MDP20	8.25	15.5	4.72
GPR241	DEL20	8.28	15.06	5.31	MDP20	8.04	16.07	4.77
GPR247	DEL20	9.14	14.19	5.27	MDP20	9.18	13.45	5.4
GPR248	DEL20	8.77	14.3	5.12	MDP20	9.34	15.06	5.21
GPR251	DEL20	8.57	13.96	4.85	MDP20	9.11	15.61	5.51
GPR255	DEL20	8.87	14.44	4.98	MDP20	8.78	14.44	4.94
GPR258	DEL20	8.93	15.57	5.22	MDP20	9.19	14.47	5.06
GPR259	DEL20	8.28	17.97	4.75	MDP20	8.14	15.28	4.52
GPR260	DEL20	8.55	14.21	5.08	MDP20	9.24	14.46	5.4
GPR262	DEL20	9.18	16.6	5.16	MDP20	8.71	14.58	4.71
GPR263	DEL20	8.78	16.43	4.91	MDP20	9.17	15.06	5.09
GPR267	DEL20	8.46	14.36	4.93	MDP20	9.06	14.36	5.31
GPR270	DEL20	8.01	13.29	4.71	MDP20	8.12	15.58	4.31
GPR283	DEL20	8.6	14.21	5.43	MDP20	9.36	15.69	5.35
GPR290	DEL20	8.73	13.69	4.98	MDP20	8.88	13.86	4.99
GPR292	DEL20	8.56	13.4	4.83	MDP20	8.97	14.02	5.08
GPR293	DEL20	8.62	14.49	5.17	MDP20	8.14	14.11	4.46
GPR296	DEL20	8.8	14.15	5.12	MDP20	8.13	14.93	4.62
GPR298	DEL20	8.46	14.7	4.79	MDP20	8.24	15.47	4.53
GPR301	DEL20	8.56	12.19	5.28	MDP20	8.88	13.29	5.09
GPR303	DEL20	7.56	13.45	4.64	MDP20	8.04	13.94	4.47
GPR305	DEL20	8.62	12.59	5.45	MDP20	8.24	13.73	4.95
GPR308	DEL20	9.47	14.31	5.63	MDP20	9.27	13.73	5.3
GPR310	DEL20	8.52	14.66	5.25	MDP20	8.35	13.77	4.93
GPR312	DEL20	8.36	16.57	4.88	MDP20	8.21	14.94	4.61
GPR313	DEL20	8.64	15.18	5.03	MDP20	8.5	16.73	4.63
GPR314	DEL20	8	14.98	4.87	MDP20	8.08	15.76	4.39
GPR315	DEL20	6.45	12.48	3.29	MDP20	6.7	15.65	3.59
GPR319	DEL20	8.76	16.07	5.3	MDP20	8.5	15	4.9
GPR321	DEL20	7.81	13.58	4.91	MDP20	8.28	16.01	4.92

Genotype	Environment	KLBC	KLAC	LBR	Environment	KLBC	KLAC	LBR
GPR324	DEL20	8.42	13.85	5.09	MDP20	9.02	16.03	5.1
GPR329	DEL20	8.76	15.4	5.2	MDP20	9.04	15.48	5.44
GPR331	DEL20	8.63	15.33	5.58	MDP20	8.79	16.67	5.13
GPR334	DEL20	8.34	14.46	4.86	MDP20	9.17	14.72	5.5
GPR335	DEL20	8.39	14.07	5.05	MDP20	8.7	15.18	4.93
GPR338	DEL20	7.87	14.42	4.79	MDP20	8.8	15.17	4.92
GPR339	DEL20	8.07	14.84	4.95	MDP20	8.13	NA	4.61
GPR341	DEL20	8.41	17.55	4.79	MDP20	8.93	17.02	5.09
GPR360	DEL20	7.7	14.05	4.52	MDP20	7.84	14.81	4.38
GPM4	DEL20	6.85	11.66	4.39	MDP20	6.74	11.4	4.39
GPM7	DEL20	7.38	11.43	4.86	MDP20	7.16	12.66	4.56
GPM11	DEL20	6.81	12.24	4.65	MDP20	6.81	12.06	4.72
GPM16	DEL20	7.1	12.71	4.54	MDP20	7.04	13.78	4.98
GPM20	DEL20	7.62	13.77	5.31	MDP20	7.89	14.17	5.5
GPM21	DEL20	7.51	12.44	5.43	MDP20	7.44	NA	5.16
GPM22	DEL20	7.43	12.69	5.16	MDP20	7.92	12.46	5.62
GPM23	DEL20	7.03	12.77	3.56	MDP20	7.63	12.36	5.46
GPM25	DEL20	7.76	15.27	4.9	MDP20	7.52	15.12	4.26
GPM26	DEL20	7.54	14.26	4.6	MDP20	7.54	14.3	4.51
GPM27	DEL20	8.09	13.46	5.14	MDP20	8.13	14.31	5.42
GPM28	DEL20	7.89	13.82	5.3	MDP20	7.26	14.32	4.17
GPM29	DEL20	8.33	13.24	5.32	MDP20	7.82	13.99	4.7
GPM30	DEL20	8.06	13.33	5.18	MDP20	8.1	13.98	5.39
GPM33	DEL20	8.21	13.27	5.37	MDP20	8.01	14.55	5.01
GPM35	DEL20	7.38	12.14	5.33	MDP20	7.43	14.03	4.61
GPM36	DEL20	7.18	12.76	4.82	MDP20	7.67	13.87	4.91
GPM37	DEL20	7.98	14.91	5.36	MDP20	7.81	14.26	5.07
GPM40	DEL20	7.61	13.89	5.01	MDP20	7.74	13.87	4.92
GPM45	DEL20	7.87	12.94	4.72	MDP20	8.03	13.1	4.76
GPM48	DEL20	8.06	12.39	4.71	MDP20	8.19	13.12	5.16
GPM49	DEL20	7.74	13.15	5.26	MDP20	8.08	15.57	4.76
GPM53	DEL20	7.84	13.7	4.85	MDP20	8.15	15.87	5.05
GPM55	DEL20	7.89	12.73	4.81	MDP20	8.07	NA	4.8
GPM60	DEL20	7.88	14.31	4.85	MDP20	7.91	15.76	4.79
GPM61	DEL20	7.75	14.53	4.75	MDP20	7.94	17.68	4.74
GPM71	DEL20	7.97	14.2	5.01	MDP20	8.17	15.65	5.15
GPM77	DEL20	7.41	13.5	4.73	MDP20	7.83	13.97	4.67
GPM81	DEL20	7.97	13.31	5.18	MDP20	8.26	13.7	4.91
GPM82	DEL20	8.26	13.52	5.11	MDP20	8.01	13.62	4.85

Genotype	Environment	KLBC	KLAC	LBR	Environment	KLBC	KLAC	LBR
GPM83	DEL20	7.91	12.95	5.12	MDP20	7.23	14.09	4.35
GPM85	DEL20	7.63	11.53	4.92	MDP20	7.51	12.76	4.77
GPM86	DEL20	6.42	12.56	3.89	MDP20	6.56	13.13	3.73
GPM87	DEL20	6.94	13.02	3.84	MDP20	7.55	14.03	4.85
GPM88	DEL20	6.3	12.01	3.63	MDP20	6.63	12.64	3.61
GPM91	DEL20	8.45	12.02	5.92	MDP20	8.13	12.31	5.56
GPM93	DEL20	6.44	11.58	3.58	MDP20	7	13.89	4.37
GPM94	DEL20	6.54	12.07	4.67	MDP20	6.58	12.54	4.45
GPM98	DEL20	7.72	10.77	4.77	MDP20	8.05	13.37	5.81
GPM100	DEL20	7.91	12.53	5.2	MDP20	8.01	12.71	5.68
GPM101	DEL20	7.56	12.17	5.23	MDP20	8.25	13.53	5.95
GPM105	DEL20	8.09	12.14	5.67	MDP20	7.92	13.22	5.61
GPM106	DEL20	8.03	13.1	5.59	MDP20	7.99	13.05	5.5
GPM109	DEL20	6.99	12.76	4.43	MDP20	6.94	10.79	4.29
GPM113	DEL20	6.98	13.43	4.41	MDP20	6.76	11.05	4.2
GPM114	DEL20	6.93	12.2	4.35	MDP20	6.4	10.81	4.06
GPM115	DEL20	7.01	12.38	4.35	MDP20	7.1	12.91	4.26
GPM117	DEL20	7.23	12.47	4.79	MDP20	7.89	12.06	5.09
GPM118	DEL20	7.47	11.95	5.24	MDP20	7.13	14.07	4.42
GPM124	DEL20	7.18	11.35	5.13	MDP20	7.63	13.36	5.37
GPM127	DEL20	7.52	12.43	5.21	MDP20	7.35	13.4	5.06
P1401	DEL20	7.79	15.39	5.72	MDP20	7.69	15.97	5.75
ABL19	DEL20	8.32	14.49	4.78	MDP20	8.51	15.42	4.58
PB1509	DEL20	8.23	16.2	4.83	MDP20	8.84	16.92	4.97
P6B	DEL20	5.85	9.39	3.91	MDP20	6.15	9.76	3.81
PRR78	DEL20	7.85	13.63	4.85	MDP20	7.98	13.89	4.44
GPR3	KNL20	7.97	14	4.6	RKR20	7.89	14.25	4.47
GPR4	KNL20	8.01	13.09	4.75	RKR20	7.96	14.3	4.54
GPR7	KNL20	6.18	10.42	3.74	RKR20	6.58	11.29	3.04
GPR8	KNL20	7.79	13.44	4.72	RKR20	7.46	14.48	4.24
GPR19	KNL20	8.91	16.7	5.17	RKR20	8.67	17.39	5.03
GPR21	KNL20	6.85	11.04	3.78	RKR20	7.02	13.33	3.79
GPR23	KNL20	8.11	14.09	5.19	RKR20	7.84	15.25	4.29
GPR24	KNL20	8.02	15.69	4.53	RKR20	8.52	16.64	4.41
GPR32	KNL20	8.33	15.39	4.79	RKR20	8.32	16.81	4.48
GPR35	KNL20	8.03	14.42	4.77	RKR20	8.17	15.46	4.5
GPR37	KNL20	7.69	13.49	4.58	RKR20	8	14.59	4.42
GPR38	KNL20	8.88	16.62	5.29	RKR20	8.68	15.03	4.7
GPR39	KNL20	8.65	15.36	5.18	RKR20	8.42	15.59	4.46

Genotype	Environment	KLBC	KLAC	LBR	Environment	KLBC	KLAC	LBR
GPR42	KNL20	7.91	14.12	4.86	RKR20	8.08	15.78	4.58
GPR43	KNL20	5.92	9.69	3.18	RKR20	5.73	10.38	3.05
GPR45	KNL20	7.49	13.79	3.68	RKR20	7.29	14.34	3.58
GPR47	KNL20	8.19	15.97	4.9	RKR20	8	14.62	4.49
GPR52	KNL20	8.13	14.96	5.01	RKR20	8.25	16.39	4.39
GPR60	KNL20	8.33	14.51	4.89	RKR20	8.08	15.14	4.33
GPR62	KNL20	7.85	14.6	4.69	RKR20	8.19	15.49	4.4
GPR67	KNL20	8.3	14.05	5	RKR20	8.27	16.26	4.59
GPR70	KNL20	7.93	14.78	4.74	RKR20	8.16	14.74	4.44
GPR74	KNL20	7.51	14.62	4.47	RKR20	7.95	13.13	4.86
GPR77	KNL20	7.63	14.15	4.37	RKR20	8.26	15.19	4.45
GPR78	KNL20	7.9	14.78	4.61	RKR20	8.26	15.24	4.32
GPR80	KNL20	7.96	14.07	5.86	RKR20	8.49	15.68	4.3
GPR82	KNL20	7.89	13.84	4.65	RKR20	8.23	14.81	4.45
GPR86	KNL20	7.76	14.23	4.72	RKR20	8.18	15.06	4.41
GPR87	KNL20	8	15.04	4.69	RKR20	7.9	14.86	4.24
GPR92	KNL20	8.2	14.78	4.79	RKR20	7.92	12.73	4.5
GPR96	KNL20	7.79	14.37	4.66	RKR20	8.63	15.04	4.75
GPR100	KNL20	7.87	14.13	4.98	RKR20	8.16	15.47	4.61
GPR102	KNL20	8.06	15.22	4.77	RKR20	8.43	15.82	4.62
GPR104	KNL20	8.06	15.81	4.88	RKR20	8.28	14.57	4.59
GPR106	KNL20	8.01	14.47	4.65	RKR20	8.29	15.28	4.81
GPR111	KNL20	8.48	16.32	5.01	RKR20	8.26	16.84	4.88
GPR112	KNL20	7.96	16.02	4.81	RKR20	7.97	14.34	4.51
GPR113	KNL20	8.18	16.26	5.15	RKR20	7.93	15.45	4.6
GPR114	KNL20	7.57	15.24	4.78	RKR20	7.87	15.29	4.03
GPR115	KNL20	8.73	16.26	5.21	RKR20	8.2	15.39	4.63
GPR117	KNL20	8.57	17.15	5.33	RKR20	8.08	16.29	4.87
GPR118	KNL20	8.56	16.3	4.95	RKR20	8.39	16.97	4.57
GPR119	KNL20	8.63	15.86	5.32	RKR20	8.52	16.77	4.59
GPR120	KNL20	7.7	13.41	4.73	RKR20	7.83	14.95	4.31
GPR131	KNL20	7.63	15.24	5.02	RKR20	7.62	14.16	4.6
GPR136	KNL20	7.6	13.95	4.9	RKR20	8.02	14.73	4.22
GPR142	KNL20	7.79	14.29	4.56	RKR20	8.26	15.31	4.51
GPR144	KNL20	7.98	14.76	4.72	RKR20	7.93	13.89	4.6
GPR146	KNL20	7.85	14.67	4.88	RKR20	7.82	14.62	4.37
GPR147	KNL20	7.73	14.36	4.6	RKR20	8.25	14.08	4.29
GPR148	KNL20	7.99	14.88	4.62	RKR20	8.16	14.8	4.53
GPR149	KNL20	7.87	14.47	4.78	RKR20	8.36	14.97	4.6

Genotype	Environment	KLBC	KLAC	LBR	Environment	KLBC	KLAC	LBR
GPR150	KNL20	8.15	14.99	4.9	RKR20	8.03	14.98	4.5
GPR151	KNL20	7.71	14.22	4.61	RKR20	8.31	14.33	4.4
GPR155	KNL20	8.07	14.88	4.66	RKR20	8.7	15.65	4.8
GPR157	KNL20	7.73	15.54	4.36	RKR20	8.28	14.56	4.57
GPR160	KNL20	7.85	14.92	4.76	RKR20	7.99	15.11	4.21
GPR164	KNL20	7.2	14.48	5.24	RKR20	7.65	14.77	4.9
GPR166	KNL20	7.21	12.41	4.82	RKR20	7.05	12.23	4.07
GPR173	KNL20	7.23	13.55	4.52	RKR20	7.81	15.11	4.3
GPR175	KNL20	7.68	15.35	4.81	RKR20	8.05	14.87	4.49
GPR176	KNL20	8	17.06	5.01	RKR20	8.38	16.77	4.64
GPR177	KNL20	7.86	15.25	4.63	RKR20	8.3	16.91	4.73
GPR194	KNL20	6.71	11.58	4.18	RKR20	7.23	11.95	3.97
GPR195	KNL20	9.22	14.59	5.49	RKR20	9.05	15.54	5.03
GPR196	KNL20	9.24	16.88	5.6	RKR20	9.28	17.24	4.93
GPR202	KNL20	8.34	15.83	4.74	RKR20	8.57	16.87	4.65
GPR206	KNL20	8.11	14.86	4.75	RKR20	8.3	16.1	4.31
GPR209	KNL20	8.28	15.28	4.75	RKR20	8.2	15.18	4.45
GPR213	KNL20	8.91	16.35	4.72	RKR20	8.01	14	4.45
GPR216	KNL20	8.41	13.79	5.08	RKR20	8.15	13.76	4.51
GPR222	KNL20	9.05	15.27	5.56	RKR20	8.96	15.07	4.97
GPR231	KNL20	8	13.55	5.4	RKR20	8.21	15.72	4.51
GPR234	KNL20	8.63	14.64	5.56	RKR20	8.54	15.59	4.92
GPR237	KNL20	8.72	15.23	5.8	RKR20	8.69	15.61	4.89
GPR238	KNL20	7.96	13.23	5.68	RKR20	8.13	14.2	4.59
GPR239	KNL20	7.92	14.81	5.24	RKR20	8.25	14.45	4.57
GPR240	KNL20	8.29	15.87	4.99	RKR20	7.83	14.94	4.31
GPR241	KNL20	7.26	14.13	4.81	RKR20	8.53	16.86	4.91
GPR247	KNL20	8.54	15.6	5.14	RKR20	8.51	14.4	4.42
GPR248	KNL20	8.68	15.22	5.07	RKR20	8.67	14.59	4.76
GPR251	KNL20	9	14.04	5	RKR20	8.5	14.57	4.39
GPR255	KNL20	8.77	14.81	5.01	RKR20	8.45	14.93	4.43
GPR258	KNL20	8.83	14.74	4.9	RKR20	8.67	15.71	4.7
GPR259	KNL20	8.03	16.29	4.5	RKR20	8.14	14.38	4.4
GPR260	KNL20	8.67	13.82	4.9	RKR20	8.91	14.98	4.79
GPR262	KNL20	9.1	15.64	5.02	RKR20	8.61	15.14	4.62
GPR263	KNL20	8.56	14.93	4.64	RKR20	8.54	16.83	4.57
GPR267	KNL20	8.85	13.18	4.83	RKR20	8.7	15.36	4.56
GPR270	KNL20	8.12	14.79	4.42	RKR20	8.15	14.81	4.14
GPR283	KNL20	7.56	14.05	4.48	RKR20	9.07	14.6	4.88

Genotype	Environment	KLBC	KLAC	LBR	Environment	KLBC	KLAC	LBR
GPR290	KNL20	8.56	15.21	5.09	RKR20	8.82	15.56	4.48
GPR292	KNL20	8.83	14.6	5.03	RKR20	8.71	14.99	4.64
GPR293	KNL20	8.79	13.64	5.13	RKR20	8.69	14.23	4.49
GPR296	KNL20	8.94	14.22	5.09	RKR20	8.34	13.74	4.4
GPR298	KNL20	7.83	14.48	4.44	RKR20	8.39	15.96	4.7
GPR301	KNL20	7.48	13.41	4.52	RKR20	8.3	13.95	4.51
GPR303	KNL20	8.06	13.45	4.86	RKR20	8.33	12.68	4.81
GPR305	KNL20	7.73	13.64	4.97	RKR20	7.92	13.56	4.58
GPR308	KNL20	9	13.99	5.6	RKR20	8.12	13.63	4.44
GPR310	KNL20	8.07	13.86	5.07	RKR20	8.33	13.84	4.72
GPR312	KNL20	7.76	15.79	4.68	RKR20	8.79	15.24	4.73
GPR313	KNL20	8.63	17.4	5.05	RKR20	8.46	15.98	4.49
GPR314	KNL20	8.06	14.68	4.43	RKR20	7.87	16.24	4.41
GPR315	KNL20	6.72	11.71	3.5	RKR20	7.88	14.8	4.73
GPR319	KNL20	8.5	14.18	4.87	RKR20	8.51	17.58	4.41
GPR321	KNL20	8.27	15.07	4.95	RKR20	8.36	16.03	4.47
GPR324	KNL20	8.36	16.15	5.09	RKR20	8.39	15.34	4.78
GPR329	KNL20	8.77	17.02	5.63	RKR20	8.7	15.81	4.66
GPR331	KNL20	8.44	14.67	5.09	RKR20	8.88	15.94	4.92
GPR334	KNL20	8.1	14.09	4.94	RKR20	8.43	15.44	4.35
GPR335	KNL20	8.39	14.59	5.22	RKR20	7.65	14.78	4.23
GPR338	KNL20	7.82	14.71	4.68	RKR20	8.26	13.34	4.51
GPR339	KNL20	8.19	15.28	4.74	RKR20	8.21	16.54	4.54
GPR341	KNL20	8.21	16.5	4.94	RKR20	8.52	16.25	4.83
GPR360	KNL20	7.82	15.88	4.6	RKR20	8.09	15.28	4.35
GPM4	KNL20	5.73	10.91	3.56	RKR20	7.02	11.82	3.95
GPM7	KNL20	7.87	10.07	5.31	RKR20	7.65	12.43	4.4
GPM11	KNL20	6.05	10.83	4.04	RKR20	7.22	12.44	4.35
GPM16	KNL20	6.37	11.79	4.14	RKR20	6.49	12.14	3.54
GPM20	KNL20	7.29	12.94	4.65	RKR20	7.81	13.03	4.47
GPM21	KNL20	7.37	12.51	5.09	RKR20	8.07	13.34	5.4
GPM22	KNL20	7.25	12.26	4.94	RKR20	7.74	12.74	4.96
GPM23	KNL20	7.03	12.13	5.03	RKR20	7.52	12.16	4.99
GPM25	KNL20	7.72	16.34	4.67	RKR20	8.42	17.28	4.68
GPM26	KNL20	7.58	15.11	4.49	RKR20	8.08	14.89	5.18
GPM27	KNL20	8.07	13.79	5.52	RKR20	7.71	14.25	4.47
GPM28	KNL20	7.58	11.39	5.27	RKR20	7.7	13.78	4.51
GPM29	KNL20	8.19	12.83	5.54	RKR20	8.44	14.45	4.91
GPM30	KNL20	7.86	11.82	5.55	RKR20	8.28	12.95	5.28

Genotype	Environment	KLBC	KLAC	LBR	Environment	KLBC	KLAC	LBR
GPM33	KNL20	8.23	13.72	5.74	RKR20	8.09	14.78	5.26
GPM35	KNL20	7.19	12.13	4.92	RKR20	7.93	11.98	4.45
GPM36	KNL20	7.35	12.05	4.85	RKR20	8.06	13.54	4.7
GPM37	KNL20	7.86	13.28	5.19	RKR20	7.76	14.6	4.56
GPM40	KNL20	7.39	13.96	4.97	RKR20	7.76	13.53	4.6
GPM45	KNL20	7.61	12.31	5.62	RKR20	7.52	13.02	4.42
GPM48	KNL20	8.17	12.57	4.82	RKR20	8.18	13.24	4.35
GPM49	KNL20	8.49	14.1	5.19	RKR20	8.46	14.59	4.74
GPM53	KNL20	8.56	13.49	5.21	RKR20	8.25	13.9	4.77
GPM55	KNL20	8.67	16.14	5.39	RKR20	8.25	14.87	4.59
GPM60	KNL20	8.64	15.34	5.28	RKR20	8.7	13.38	4.47
GPM61	KNL20	8.19	15.45	4.99	RKR20	8.43	14.34	4.62
GPM71	KNL20	8.4	14.68	5.17	RKR20	8.29	15.25	4.13
GPM77	KNL20	7.85	13.83	5.09	RKR20	8.76	12.67	4.98
GPM81	KNL20	8.5	13.55	5.54	RKR20	8.7	13.4	4.77
GPM82	KNL20	8.52	13.21	5.14	RKR20	7.69	13.65	4.22
GPM83	KNL20	7.82	12.27	4.77	RKR20	7.86	13.12	4.5
GPM85	KNL20	7.75	13.21	4.83	RKR20	7.55	12.21	4.39
GPM86	KNL20	6.87	11.83	4.31	RKR20	6.47	12.22	3.76
GPM87	KNL20	7.45	12.02	4.14	RKR20	6.96	13.5	3.81
GPM88	KNL20	6.65	11.25	3.78	RKR20	6.57	13.18	3.76
GPM91	KNL20	8.14	11.58	5.74	RKR20	8.61	11.82	5.73
GPM93	KNL20	7.35	10.29	4.21	RKR20	6.79	12.33	3.89
GPM94	KNL20	6.82	12.56	4.74	RKR20	7.02	12.2	4.11
GPM98	KNL20	8.86	13.7	5.08	RKR20	8.06	12.72	4.7
GPM100	KNL20	8.76	12.57	5.92	RKR20	7.92	13	4.9
GPM101	KNL20	8.17	12.11	5.37	RKR20	8.12	12.97	5.36
GPM105	KNL20	8.72	13.08	5.85	RKR20	7.97	12.99	4.88
GPM106	KNL20	NA	NA	NA	RKR20	7.99	12.11	5.27
GPM109	KNL20	7.06	13.19	4.39	RKR20	6.95	13.71	4.24
GPM113	KNL20	6.86	13.11	4.21	RKR20	7.06	13.35	4.16
GPM114	KNL20	7.35	13.49	4.54	RKR20	7.24	12.96	4.35
GPM115	KNL20	7.32	13.05	4.46	RKR20	7.16	13.53	4.45
GPM117	KNL20	7.89	13.57	5.13	RKR20	7.84	13.29	4.81
GPM118	KNL20	8.13	13.66	5.19	RKR20	7.68	13.45	4.87
GPM124	KNL20	8.02	13.17	5.61	RKR20	7.74	12.98	5.05
GPM127	KNL20	7.83	13.2	5.09	RKR20	7.72	12.72	4.85
P1401	KNL20	7.69	15.4	5.32	RKR20	7.91	16.93	5.01
ABL19	KNL20	7.97	13.68	4.68	RKR20	8.15	15.65	4.37

Genotype	Environment	KLBC	KLAC	LBR	Environment	KLBC	KLAC	LBR
PB1509	KNL20	8.25	15.97	5.13	RKR20	8.18	17.16	4.5
P6B	KNL20	5.92	10.12	3.88	RKR20	6	9.96	3.63
PRR78	KNL20	7.4	13.88	4.49	RKR20	7.65	13.48	4.35

KLBC, Kernel length before cooking in mm; LBR, Length-Breadth Ratio; KLAC, Kernel length after cooking in mm.

Supplementary Table 6: Pooled BLUEs of the selected individuals which carry superior allelic combinations for DFF, PHT, PL, KLBC and LBR

Genotypes	DFF	PHT	PL	KLBC	LBR
ABL19	89.10	106.59	28.66	8.24	4.60
GPR313	85.16	110.30	27.97	8.56	4.80
GPR283	86.60	107.17	28.53	8.65	5.04
GPR87	87.99	105.27	27.65	8.25	4.74
GPR104	88.02	110.40	27.79	8.13	4.76
GPR335	90.86	101.74	28.11	8.28	4.86
GPR96	88.44	109.77	28.68	8.38	4.94
GPR82	88.86	104.38	27.51	8.14	4.67
GPR310	85.46	107.66	28.14	8.32	4.99
GPR263	90.85	100.85	28.13	8.76	4.80
GPR262	90.44	104.12	28.67	8.90	4.88
GPR24	92.43	108.07	28.92	8.35	4.65
GPR270	87.43	108.68	28.19	8.10	4.40
GPR142	90.58	108.49	27.56	8.03	4.69
GPR338	90.56	103.88	29.04	8.19	4.72
GPR248	91.42	113.16	30.84	8.87	5.04
GPR267	89.99	101.22	28.22	8.77	4.91
GPR150	89.28	107.46	28.12	8.14	4.78
GPR149	89.02	108.02	28.59	8.23	4.92
GPR255	89.88	107.35	28.62	8.71	4.84
GPR148	89.86	110.18	28.36	8.12	4.76
GPM98	102.83	109.14	29.48	8.17	5.09
GPR292	89.99	103.14	28.48	8.77	4.90
GPR251	88.14	108.86	29.26	8.80	4.94
GPR80	88.28	106.72	27.79	8.26	4.99
GPR259	85.86	97.83	26.07	8.15	4.54
GPR312	84.72	112.14	27.90	8.28	4.73
GPM105	90.89	106.09	30.02	8.18	5.50
GPR290	88.88	105.99	27.52	8.75	4.89
GPR19	93.13	106.66	28.54	8.48	5.04
GPR319	88.88	110.18	28.00	8.57	4.87
GPR37	83.46	99.86	28.85	8.07	4.65
GPR296	89.59	105.78	28.39	8.55	4.81
GPR3	90.29	107.25	27.70	7.97	4.78
GPR70	87.28	108.09	27.81	8.04	4.75
GPR92	89.43	108.81	27.90	8.11	4.75
GPR52	87.44	105.87	24.77	8.37	4.88
GPR314	89.15	110.86	28.02	8.01	4.53

Genotypes	DFF	PHT	PL	KLBC	LBR
GPR195	90.43	130.14	29.80	9.11	5.24
GPR39	92.70	110.08	28.20	8.39	4.78
GPR151	90.01	108.28	27.71	7.98	4.60
GPR120	87.88	98.69	26.91	7.72	4.66
GPR86	87.72	105.93	27.94	8.17	4.70
GPR8	84.86	122.43	28.10	7.97	4.85
GPR146	89.02	108.27	27.53	8.03	4.78
GPR102	90.44	109.48	28.74	8.26	4.78
GPR164	83.30	109.72	27.32	7.81	5.18
GPR308	86.46	106.08	27.95	8.97	5.24
PB1509	83.10	100.94	26.95	8.37	4.86
GPR216	83.32	113.82	27.66	8.64	5.14
GPR247	89.58	110.44	28.94	8.84	5.06
GPR305	87.17	105.43	28.08	8.13	4.99
GPR360	87.72	103.65	27.37	7.86	4.46
GPR77	90.13	106.32	28.37	8.03	4.58
GPR222	85.89	111.18	30.39	9.07	5.42
GPR324	94.55	106.25	26.48	8.55	5.01
GPR74	89.01	109.51	28.23	7.98	4.77
GPR298	90.01	105.54	28.18	8.23	4.61
GPR321	91.83	103.22	26.77	8.18	4.81
GPR258	89.57	107.71	28.50	8.91	4.97
GPM100	97.26	110.47	31.89	8.15	5.43
GPR303	86.88	108.05	28.12	8.00	4.69
GPM106	100.40	108.92	31.89	8.00	5.45
GPR106	88.42	110.08	28.26	8.12	4.81
GPR147	89.16	108.91	28.30	8.09	4.68
GPM101	102.11	108.63	31.75	8.02	5.48
GPR293	90.28	108.31	28.23	8.56	4.81
GPR241	84.31	99.08	28.08	8.03	4.95
GPR67	88.30	106.81	27.66	8.12	4.83
GPR339	87.43	108.72	27.99	8.15	4.71
GPR78	89.15	108.76	27.98	8.05	4.59
GPR341	87.38	118.92	27.97	8.51	4.91
GPR173	86.57	112.96	26.88	7.56	4.49
GPR260	88.85	108.85	28.50	8.84	5.04
GPR100	88.86	117.13	28.48	8.19	4.82
GPR334	87.72	111.39	28.24	8.51	4.91
GPR301	87.14	106.31	27.04	8.31	4.85

2. Supplementary Figures

Supplementary Figure 1: Allelic effect of various selected stable MTAs, *viz*. **(A)** *qDFF6.1*, **(B)** *qDFF11.1*, **(C)** *qDFF8.1*, **(D)** *qDFF8.2* and **(E)** *qDFF5.1* in their detected environments for DFF

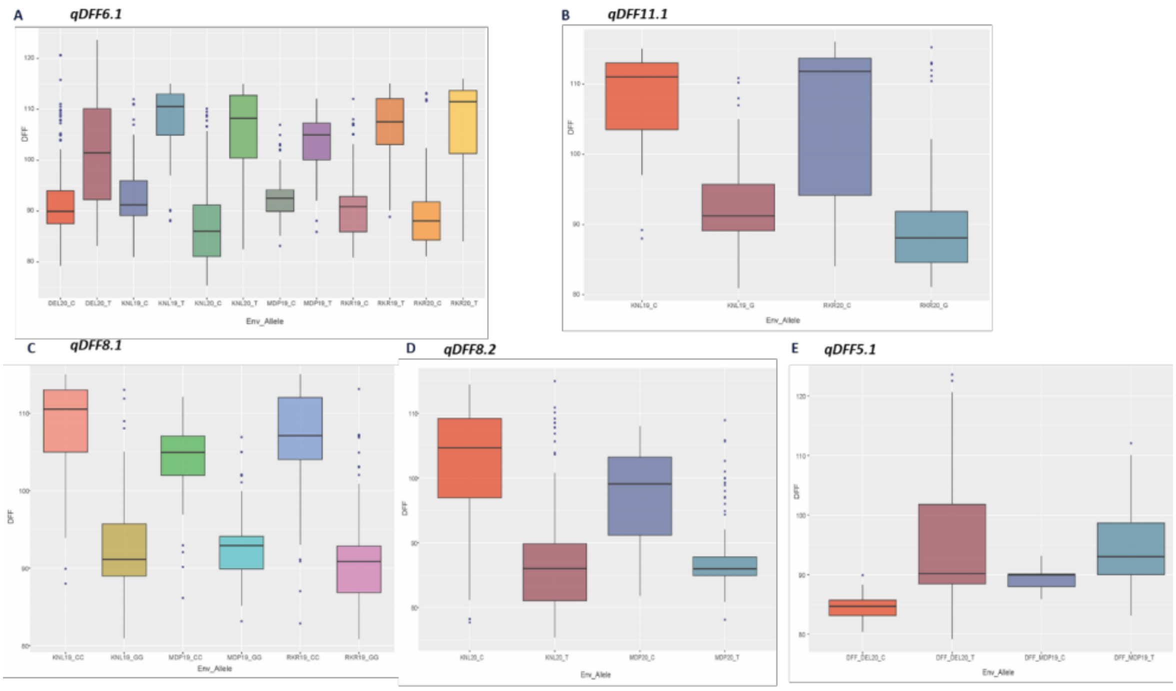

Supplementary Figure 2: Phenotypic distribution of available allelic combinations for the trait DFF. The details of order of combinations and allelic status of the MTAs are given in the attached table

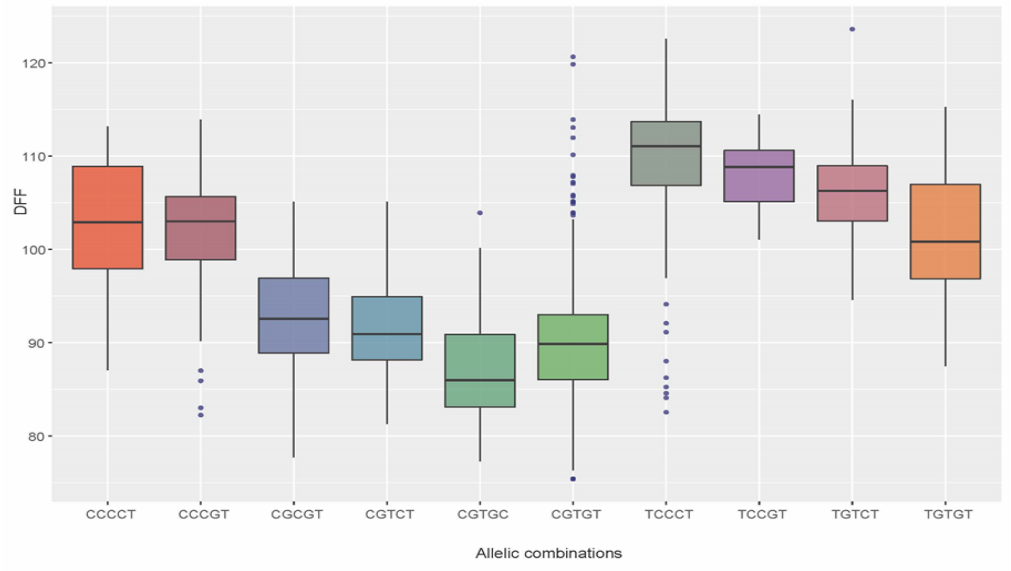

MTA	*qDFF6.1*	*qDFF8.1*	*qDFF8.2*	*qDFF11.1*	*qDFF5.1*
**Short duration allele**	C	G	T	G	C
**Alternate allele**	T	C	C	C	T

Supplementary Figure 3: Allelic effect of various selected robust MTAs, *viz*. **(A)** *qPHT1.1* **(B)** *qPHT1.2*, and **(C)** *qPHT12.1*, in their detected environments for DFF

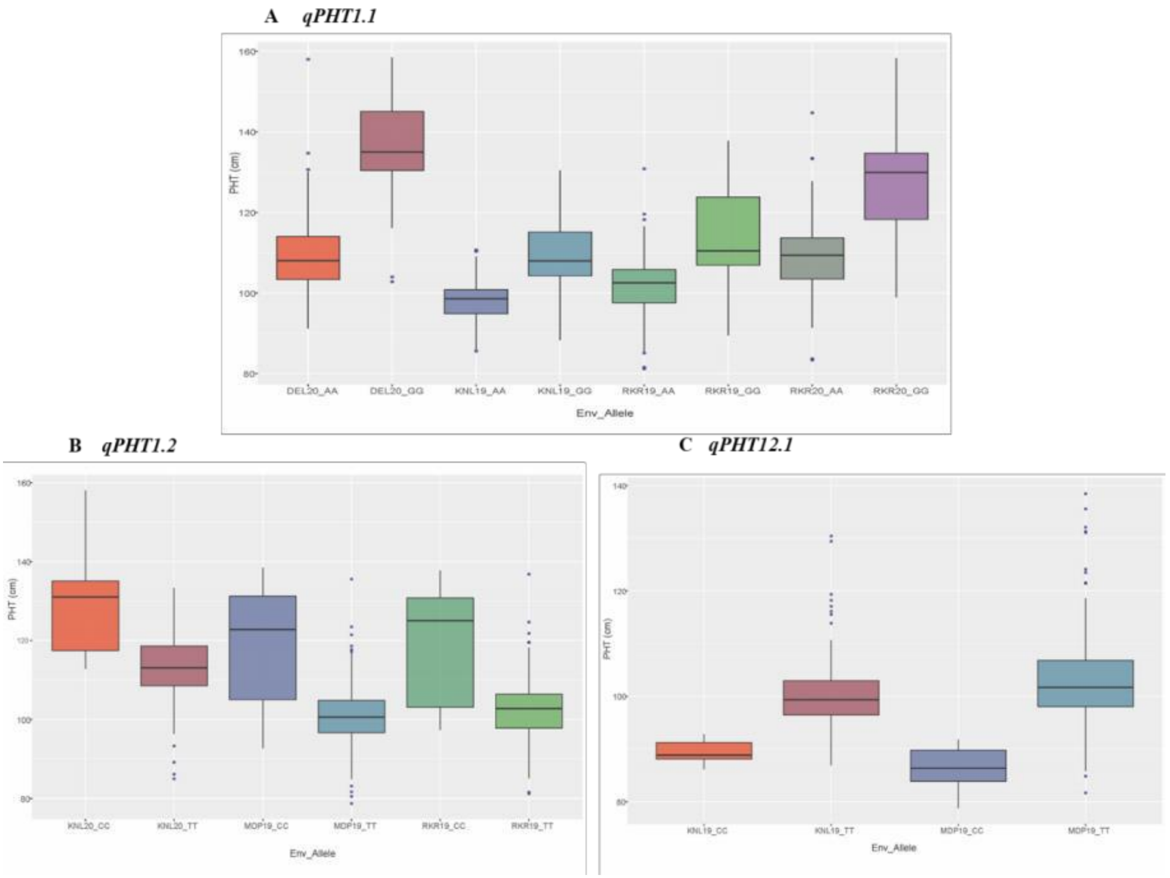

Supplementary Figure 4: Phenotypic distribution of available allelic combinations for the trait PHT. The details of order of combinations and allelic status of the MTAs are given in the attached table

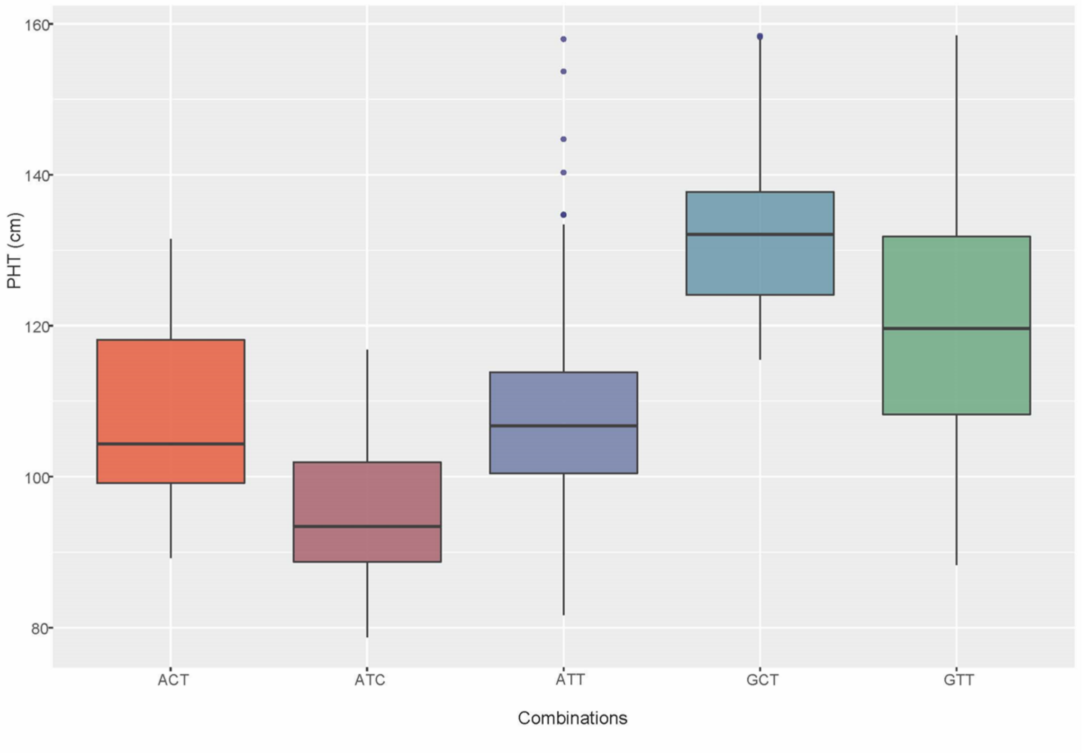

	qPHT1.1	*qPHT1.2*	*qPHT12.1*
**Semi dwarf allele**	**A**	**T**	**C**
**Alternate allele**	**G**	**C**	**T**

Supplementary Figure 5: Allelic effect of various selected robust MTAs, *viz*. **(A)** *qPL2.4* **(B)** *qPL6.1*, and **(C)** *qPL11.1*, in their detected environments for PL

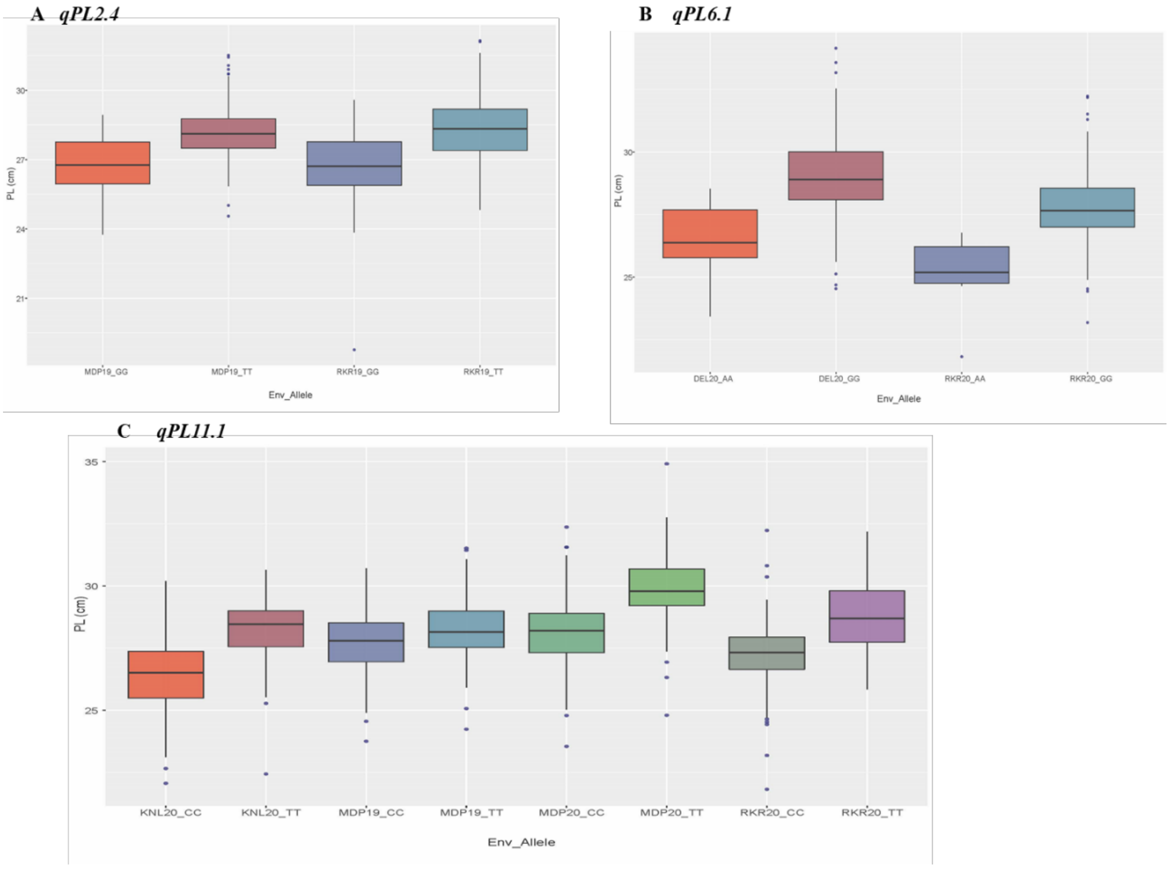

Supplementary Figure 6: Phenotypic distribution of available allelic combinations for the trait PL. The details of order of combinations and allelic status of the MTAs are given in the attached table

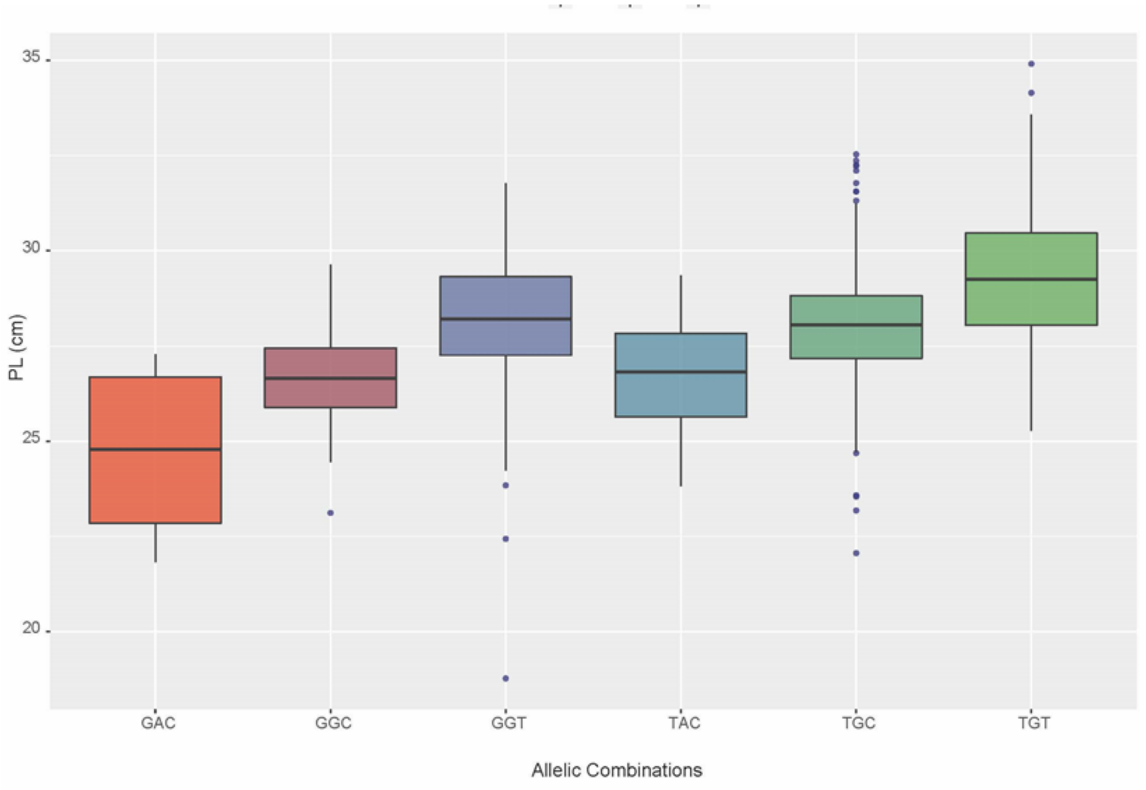

	qPL2.4	*qPL6.1*	*qPL11.1*
Favourable allele	T	G	T
Alternate allele	G	A	C

Supplementary Figure 7: Allelic effect of selected robust MTA for KLBC **(A)** *qKLBC7.1* and **(B)** *qLBR11.1* in their detected environments

A ***qKLBC7.1***

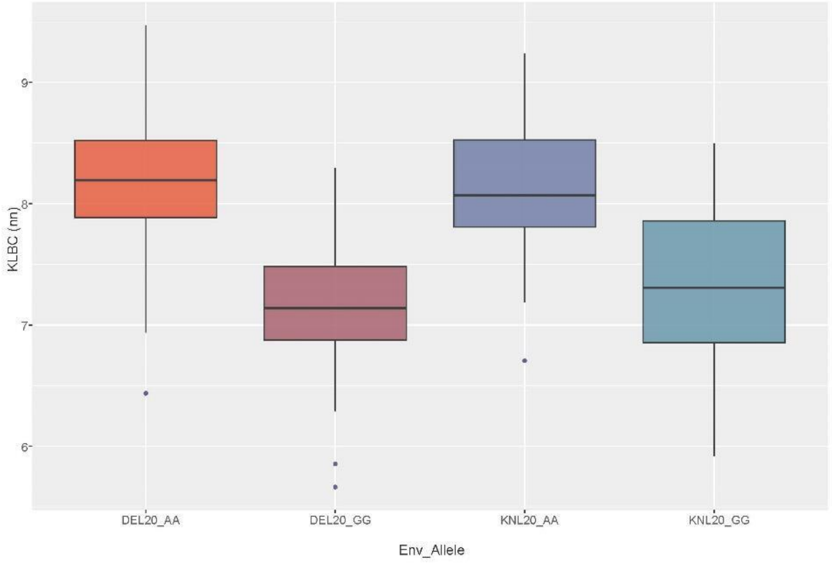

B ***qLBR11.1***

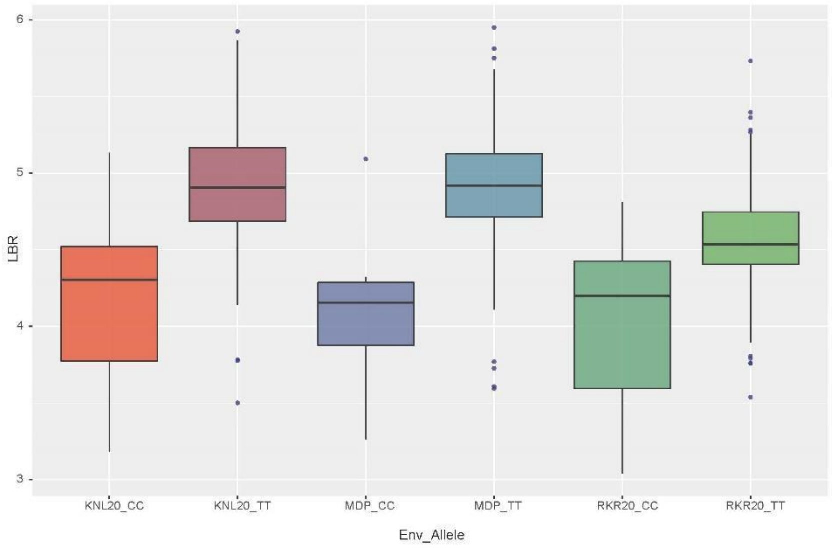

Supplement: Supplementary file 1 [file DataSheet_1.pdf]
